# Mitigating Lattice Distortion of High-Voltage LiCoO_2_ via Core-Shell Structure Induced by Cationic Heterogeneous Co-Doping for Lithium-Ion Batteries

**DOI:** 10.1007/s40820-023-01269-1

**Published:** 2023-12-11

**Authors:** Zezhou Lin, Ke Fan, Tiancheng Liu, Zhihang Xu, Gao Chen, Honglei Zhang, Hao Li, Xuyun Guo, Xi Zhang, Ye Zhu, Peiyu Hou, Haitao Huang

**Affiliations:** 1 Department of Applied Physics and Research Institute for Smart Energy, The Hong Kong Polytechnic University, Hong Kong, People’s Republic of China; 2 Department of Applied Physics, The Hong Kong Polytechnic University, Hong Kong, People’s Republic of China; 3 Institute of Nanosurface Science and Engineering, Guangdong Provincial Key Laboratory of Micro/Nano Optomechatronics Engineering, Shenzhen University, Shenzhen, People’s Republic of China; 4 School of Physics and Technology, University of Jinan, Jinan, Shandong People’s Republic of China

**Keywords:** Lithium-ion battery, LiCoO_2_, Heterogeneous co-doping, Core–shell structure, High-voltage stability

## Abstract

Inactive elemental doping is commonly used to improve the structural stability of high-voltage layered transition-metal oxide cathodes. However, the one-step co-doping strategy usually results in small grain size since the low diffusivity ions such as Ti^4+^ will be concentrated on grain boundaries, which hinders the grain growth. In order to synthesize large single-crystal layered oxide cathodes, considering the different diffusivities of different dopant ions, we propose a simple two-step multi-element co-doping strategy to fabricate core–shell structured LiCoO_2_ (CS-LCO). In the current work, the high-diffusivity Al^3+^/Mg^2+^ ions occupy the core of single-crystal grain while the low diffusivity Ti^4+^ ions enrich the shell layer. The Ti^4+^-enriched shell layer (~ 12 nm) with Co/Ti substitution and stronger Ti–O bond gives rise to less oxygen ligand holes. *In-situ* XRD demonstrates the constrained contraction of *c*-axis lattice parameter and mitigated structural distortion. Under a high upper cut-off voltage of 4.6 V, the single-crystal CS-LCO maintains a reversible capacity of 159.8 mAh g^−1^ with a good retention of ~ 89% after 300 cycles, and reaches a high specific capacity of 163.8 mAh g^−1^ at 5C. The proposed strategy can be extended to other pairs of low- (Zr^4+^, Ta^5+^, and W^6+^, etc.) and high-diffusivity cations (Zn^2+^, Ni^2+^, and Fe^3+^, etc.) for rational design of advanced layered oxide core–shell structured cathodes for lithium-ion batteries.

**Supplementary Information:**

The online version contains supplementary material available at 10.1007/s40820-023-01269-1.

## Highlights

- A simple two-step multi-element co-doping strategy is proposed to fabricate core-shell structured LiCoO_2_ based on the different diffusivities of dopant ions.
- The high diffusivity Al^3+^/Mg^2+^ ions occupy the core of single-crystal grain while the low diffusivity Ti^4+^ ions enrich the shell layer.
- In-situ XRD demonstrates the mitigated structural distortion under a high cut-off voltage of 4.6 V, resulting in a significantly improved cycling stability.

**Supplementary Information:**

The online version contains supplementary material available at 10.1007/s40820-023-01269-1.

## Graphical abstract

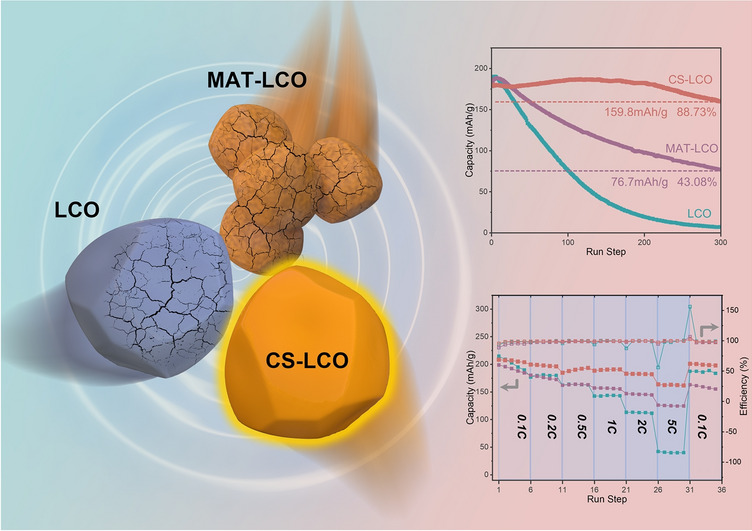

## Introduction

In recent years, the promotion of the concept of carbon neutrality has led to an increasing demand for lithium-ion batteries (LIBs) [1–3]. LIBs were firstly commercialized by Sony Corp. in 1991, in which layered oxide LiCoO_2_ (LCO) was employed as insertion/extraction cathode material in LIBs. Compared with polycrystalline cathodes, single-crystal counterpart provides several advantages in practical applications [4, 5]. Single-crystal cathodes show the special monodisperse behavior and the absence of grain boundaries, and thereby avoid the unwanted microcracks occurred in polycrystalline particles. Besides, single-crystal cathodes with lowered specific surface area and defect concentration reduce their interface side reactions and improve their structural stability. Furthermore, single-crystal cathodes typically exhibit higher mechanical strength, which can contribute to an increase in packing density. This advantage is beneficial for enhancing the volumetric energy density of LIBs [5, 6]. To satisfy the increased push of smart-mobile and fast-paced society, there is a greater need for single crystal LCO processing with higher energy density and superior structural stability. To ensure structural stability, the upper cut-off voltage of LCO is normally kept no higher than 4.5 V to avoid the transformation of LCO from O3 (octahedral triple-phase) to H1-3 (hybrid phases of octahedral O1 and O3) [7]. And the corresponding Li^+^ that can be intercalated into or de-intercalated from the LCO lattice is only ~ 65% of the total amount of Li [8]. Indeed, increasing the upper cut-off voltage could effectively obtain the higher reversible capacity, but the highly delithiated state with higher Li^+^ extraction results in a significant degradation of cycling performance, accompanied by structural collapse, irreversible phase transition, electrolyte oxidation, lattice oxygen release, and dissolution of Co [9–11].

Inactive elemental doping is commonly used to modify materials’ structure and physical properties, such as band gap, lattice structure, and charge redistribution, etc. for the optimization of their electrochemical performance [12, 13]. Different doping elements (Mg, Al, Ti, Mn, Ni, Se, Zr and Sn) are often used as dopants [14–19] to improve the electrochemical performance of LCO. Recent research by Huang et al. showed that the doping of Mg ions in the Li layer created the so-called pillar effect to stabilize the layered structure [16]. For multi-elements doping, a one-step co-doping strategy of ternary dopants was reported [19]. The synergistic effects of multi-element dopants stabilize the surface oxygen and promote the cycle stability of LCO at high-voltage of 4.6 V. Although partial success has been achieved, one-step co-doping strategy seems to bring some other problems. During the one-step co-doping process, several codoped cations (heavy and/or high valence-state cation dopants Ti^4+^, Zr^4+^, and Y^3+^, etc.) have been found to readily enrich on the grain surface and grain boundaries, and further induce the formation of polycrystalline particles due to the limited growth kinetics in various cathode materials [19–22]. These agglomerated polycrystalline particles possess increased grain boundaries and a larger specific surface area, which will inevitably react with electrolyte during charge/discharge cycling. Therefore, designing an appropriate multi-element co-doping strategy to synthesize single-crystal cathodes with high performance is still quite challenging [23–25].

The diffusivities of cations show clear dependence on cation charge and radius [20, 21, 26, 27]. In this work, considering the large variation in diffusivity of different dopant ions, we propose a novel two-step co-doping strategy to dope Ti/Mg/Al into LCO, a model layered oxide cathode material. A core–shell structured single-crystal LCO (CS-LCO) cathode material is obtained, where the high-diffusivity Al^3+^/Mg^2+^ ions occupy the core of single-crystal grains while the low diffusivity Ti^4+^ ions just enrich the shell layer of each grain. The Ti^4+^-enriched shell layer with Co/Ti substitution and stronger Ti–O bond, reduce the number of oxygen ligand holes and enhance the oxygen stability. *In-situ* XRD reveals that the CS-LCO exhibits mitigated phase transition from O3 to H1-3, resulting in reduced contraction of *c*-axis and structural distortion. Under a high cut-off voltage of 4.6 V, the CS-LCO maintains a reversible capacity of 159.8 mAh g^−1^ with a good retention of ~ 89% after 300 cycles, and reaches a high specific capacity of 163.8 mAh g^−1^ at 5C. The proposed strategy can also be extended to other couples of low-diffusivity cations (Zr^4+^, Ta^5+^, and W^6+^, etc.) and high-diffusivity ones (Zn^2+^, Ni^2+^, and Fe^3+^, etc.) for controllable construction of single-crystal core–shell structured cathodes that improve not only the structural stability, but also the electrochemical performance of other LIB cathodes. Our work described here provides a new insight into the fabrication of single-crystal LCO with enhanced electrochemical performance via multi-element doping for high energy–density LIB applications.

## Experimental Section

### Material Synthesis

The purchased Co_3_O_4_ (99.5%, International laboratory USA), Li_2_CO_3_ (99.5%, International laboratory USA), Al_2_O_3_ (99%, International laboratory USA), MgO (98%, International laboratory USA), and TiO_2_ (99.7%, Sigma-Aldrich) were directly used without purification.

### Material Synthesis

LCO, MAT-LCO and CS-LCO were prepared by a solid-state reaction method. For LCO, the Co_3_O_4_ and Li_2_CO_3_ were ground and sintered at 760 °C for 2 h and 1,000 °C for 12 h to obtain LCO. For MAT-LCO, the Co_3_O_4_, Al_2_O_3_, MgO, TiO_2_ and Li_2_CO_3_ were ground and sintered at 760 °C for 2 h and 1,000 °C for 12 h to obtain MAT-LCO. For CS-LCO, the Co_3_O_4_, Al_2_O_3_, MgO, and Li_2_CO_3_ were ground and firstly sintered at 760 °C for 2 h and 1,000 °C for 12 h to obtain AMLCO intermediate. The AMLCO powder was than mixed with TiO_2_ and sintered at 900 °C for 10 h to obtain CS-LCO.

### Materials Characterization

The morphology and phase of cathodes were investigated by scanning electron microscope (SEM, MAIA3 and VEGA3) and X-ray diffraction (XRD, Rigaku SmartLab). In-situ XRD patterns were obtained using a specially designed cell with the beryllium (Be) window and at a scan rate of 10° min^−1^. The microstructure of CS-LCO was studied by TEM (JEOL JEM-2100F). The surface components analysis was studied by X-Ray Photoelectron Spectroscopy (XPS, Thermo Fisher Scientific Nexsa). The specific surface area of powders was determined by Brunauer, Emmett, and Teller method (BET, ASAP 2020).

### Electrochemical Measurement

To fabricate the electrodes, 90% active material particles (LCO, MAT-LCO and CS-LCO), 5 wt% super P and 5 wt% polyvinylidene fluoride (PVdF) were dissolved in an appropriate amount of N-methyl-1,2-pyrrolidone (NMP) to prepare the slurries. Briefly, the mass loading of active oxides was 3.0 mg cm^−2^. For coin-type half cells, lithium metal was used as anode. The electrolyte was formed by 1 M LiPF_6_ dissolved in ethylene carbonate (EC) and dimethyl carbonate (DMC) (3:7 in volume). The punched electrodes have a diameter of 12 mm under a compression pressure of 2 MPa. Electrochemical tests were carried out by LAND instrument (CT-2001 A) at room temperature of 25 ℃. Galvanostatic intermittent titration technique (GITT) measurements were carried out on half cells within 3.0–4.6 V (versus Li^+^/Li). A titration current of 0.3C (1C = 200 mA g^−1^) was used for 10 min along with the relaxation time of 1 h to reach the quasi-equilibrium potential. Cyclic voltammetry (CV) and electrochemical impedance spectroscopy (EIS, 10^−2^–10^6^ Hz) were conducted by Solartron 1470E (AMETEK, USA).

For coin-type full cells, prelithiated graphite was applied as negative electrode. The capacity ratio of negative/positive electrode is about 1.2 in the designed full cells. The first charge cycle was conducted by charging the assembled cells at 0.05C to 3.9 V, 0.1C to 4.1 V and 0.2C to 4.3 V, the first discharge cycle was conducted by discharging the cells to 3.0 V at 0.1C. The subsequent charge–discharge cycle was conducted at 1C at 3.0–4.55 V.

### Density Functional Theory Calculation

The theoretical calculations were performed in the framework of density functional theory (DFT) implemented in the Vienna ab initio simulation package (VASP) [28, 29]. The exchange–correlation energy was described by the generalized gradient approximation (GGA) + U [30, 31] with the Perdew-Burke-Ernzerhof (PBE) flavor [32], in which, the Hubbard U parameters for the d-orbitals of Co were set to 3.32 eV [33]. For geometry optimization, the plane-wave cut-off energy of 500 eV was employed. The Brillouin-zone integration was performed using a Monkhorst–Pack grid of k-point sampling, and the meshes of Γ-centered 5 × 5 × 2 were used for the 2 × 2 × 1 supercell in the bulk structure LiCoO_2_. The convergence criteria for energy were set to be 10^−5^ eV and force was 0.01 eV Å^−1^. The climbing image nudged elastic band (CI-NEB) method [34] was applied to calculate the minimum diffusion energy barrier.

## Results and Discussion

**Fig. 1:**
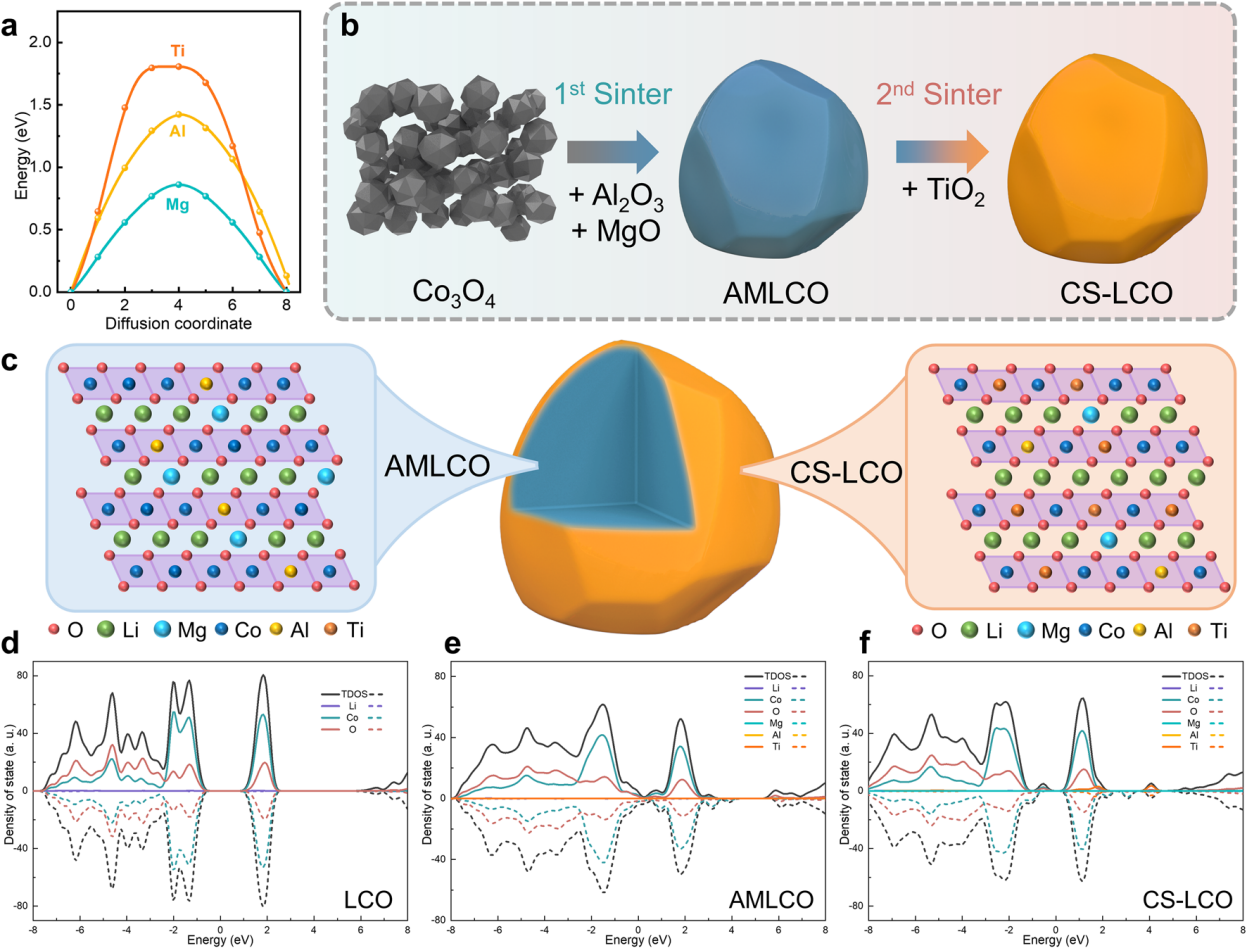
Conceptual design and preparation. **a** DFT calculation of diffusion energy barrier of Mg, Al and Ti ions along LCO interlayer. Schematic illustration of the **b** synthesis and **c** structure design of core–shell LCO with Al/Mg bulk co-doping (gray core) and gradient surface Ti doping (orange shell). Density of states (DOS) of **d** LCO, **e** AMLCO and **f** CS-LCO

For atomistic understandings, we compare the diffusion barriers of Mg, Al, and Ti ions along the interlayers using the climbing image nudged elastic band (CI-NEB) method. As shown in Fig. 1a, for Al (rAl^3+^  = 0.535 Å) and Ti (rTi^4+^  = 0.605 Å) ions diffusing along Co (rCo^3+^  = 0.545 Å) layer and Mg (rMg^2+^  = 0.72 Å) ion diffusing along Li (rLi^+^  = 0.76 Å) layer, the energy barrier follows the order of Mg (0.86 eV) < Al (1.42 eV) < Ti (1.8 eV). It is worth noting that Ti^4+^ ion exhibit the highest energy barrier among them, confirming the low diffusivity of Ti^4+^ ion in LCO. Figure 1b schematically illustrates the design and preparation of core–shell LCO (CS-LCO) via a two-step cation co-doping method, where the low diffusivity Ti^4+^ ions and high-diffusivity Al^3+^/Mg^2+^ ions are chosen as a proof of concept. In the first sintering step, Co_3_O_4_ are mixed with Li_2_CO_3_ (Li/Co = 1.1), Al_2_O_3_ (1 wt%), and MgO (1 wt%), and sintered to obtain Al^3+^/Mg^2+^ codoped LCO (AMLCO). In the second sintering step, the AMLCO powder is further mixed with TiO_2_ (1 wt%) and sintered to obtain surface-Ti^4+^-enriched core–shell structured LCO (CS-LCO). For comparison, one-step Ti /Mg/Al codoped LCO (MAT-LCO) is also prepared.

**Fig. 2:**
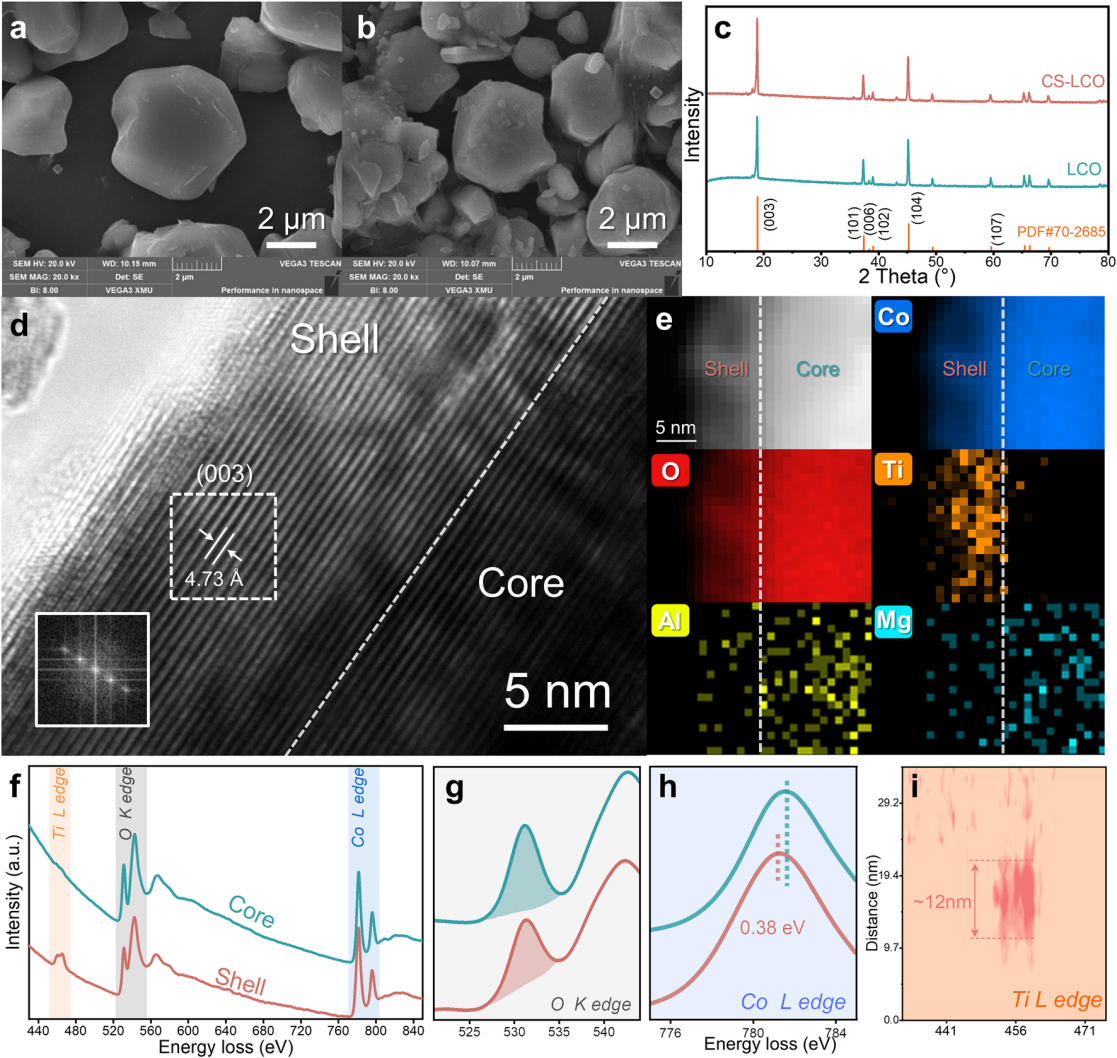
Structure characterizations. SEM image of **a** LCO and **b** CS-LCO. **c** XRD patterns of LCO and CS-LCO. **d** HRTEM image of CS-LCO. Inset indicates the FFT pattern of white rectangle. **e** Elemental distribution of O, Co, Ti, Al and Mg near the surface. **f** EELS spectra collected separately from the shell and core regions of CS-LCO, EELS line profiling of **g** O K-edge and **h** Co L-edge. **i** EELS Ti L-edge intensity mapping

SEM analyses are conducted to observe the distinctions of the cathode morphology. As shown in Figs. 2a-b and S1, the LCO and CS-LCO show micron-sized single crystallites in a size range of 2–5 µm. In contrast, MAT-LCO shows the agglomeration of primary grains with a grain size of hundreds of nanometers, which is attributed to the segregation of Ti at the grain boundaries. This segregated Ti dopant hinders the further grain growth of LCO particles into large single-crystal grains, as shown in Fig. S2, which is consistent with previous studies [19–21]. As shown in Fig. S3, the specific surface areas of the as-sintered LCO, CS-LCO, and MAT-LCO powders were measured by Brunauer–Emmett–Teller (BET), which are 0.359, 0.370 and 0.800 m^2^ g^−1^, respectively. For CS-LCO, the formation of large single crystalline particles with low surface area and high mechanical strength will help reduce side reactions and improve structural stability. XRD analysis (Fig. 2c) shows that, after co-doping, there is no detectable impurity phases in CS-LCO. All the peaks are consistent with the standard LiCoO_2_, which has a typical layered α-NaFeO_2_ structure with a space group *R-3 m* [13]. The high intensity ratio of (003)/(104) peaks and clear split of (006)/(012) peaks (Fig. S4) suggest a highly ordered layered structure of CS-LCO.

The microstructural characteristics of CS-LCO are shown in Fig. 2d. After the two-step co-doping, the surface of CS-LCO remains the layered structure with the interplanar spacing of 0.473 nm. The shell layer (~ 12 nm in thickness) exhibits a layered structure, as demonstrated by fast Fourier transform (FFT) images. Electron energy loss spectroscopy (EELS) analysis reveals a clear difference in elemental distribution between the core and the shell regions of CS-LCO. As shown in Fig. 2e, the EELS mapping reveals a higher concentration of Ti in the shell region, while Al/Mg distribute uniformly in both the core and shell region. Above results indicate the formation of a core–shell structure.

The EELS spectra show a much higher peak intensity of Ti in the shell region than that in the core region (Fig. 2f, i). Further, the EELS spectrum mapping of Ti L-edge show that the shell layer is around 12 nm thickness. Previous research has shown that the intensity of O K-edge and Co L-edge are closely related to the structural stability [35]. As shown in Fig. 2g-h, the integrated area of O K-edge in the core is larger than that in the shell, which indicates less oxygen ligand holes and enhanced lattice oxygen stability in the shell. Moreover, the peak of Co L-edge in the shell region is 0.38 eV lower than that in the core, which could be attributed to the suppressed evolution of Co (from Co^3+^ to Co^4+^) due to the substitution of Ti^4+^ in the shell [36, 37]. These results confirm that CS-LCO with Ti-enriched surface and Mg/Al-doped bulk is successfully prepared via the two-step cation co-doping method.

To further clarify the effect of Ti/Mg/Al co-doping on the electronic properties and lattice stability, the electronic density of states (DOS) of LCO before and after elemental doping are calculated by DFT. As shown in Fig.S5a, simplified structural models of LCO, Mg/Al-codoped LCO (AMLCO) and CS-LCO are constructed to analyze the influence of their intrinsic electronic conductivity. Figure 1d-f illustrates that the pristine LCO exhibits semiconducting behavior with a band gap of 1.4 eV. After Mg/Al co-doping, AMLCO exhibits a metallic character with zero band gap, due to the induced orbital hybridization between Co/O and Mg/Al dopant ions near the Fermi level. The increased DOS near the Fermi level can effectively improve the electrical conductivity. On the other hand, for CS-LCO, the induced Ti ion facilitates the charge balance of dopants, thereby preserving the semiconducting properties. The band gap of CS-LCO significantly reduces from 1.4 to 0.2 eV. These findings suggest that Ti/Mg/Al dopant ions could effectively enhance the electronic conductivity of LCO.

**Fig. 3:**
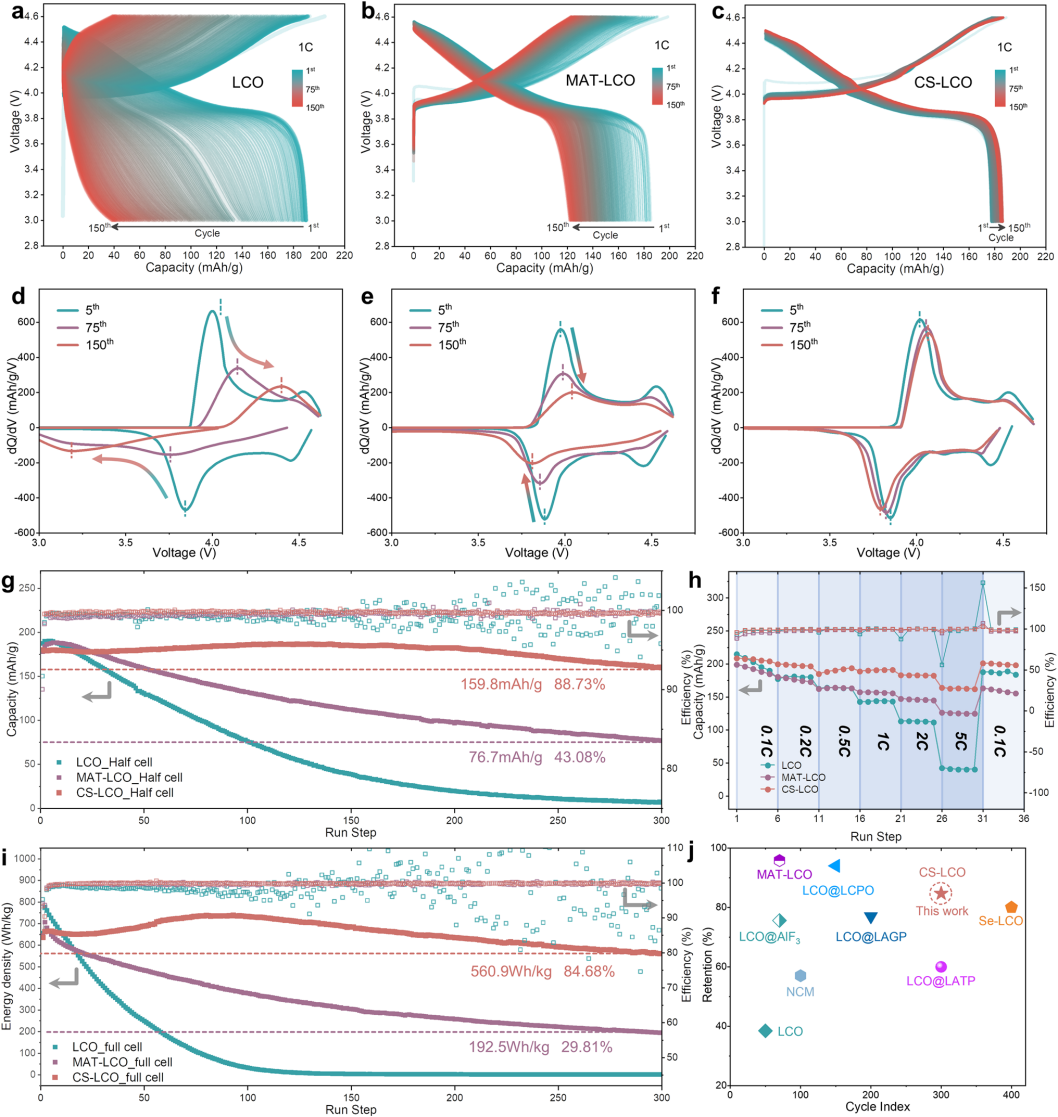
Electrochemical performance. Continuous charge/discharge curves from 1st to 150th cycles of **a** LCO, **b** MAT-LCO and **c** CS-LCO at 1C under 4.6 V. Differential capacity (dQ/dV) curves at 5th, 75th and 150th cycles of **d** LCO, **e** MAT-LCO and **f** CS-LCO. **g** Cycling stability of half-cells at 1C under 4.6 V from 1st to 300th cycles. **h** Rate stability of half-cells. **i** Cycling stability of CS-LCO//graphite full-cells at 3.0–4.55 V (equivalent to 4.6 V versus Li/Li^+^) at 1C from 1st to 300th cycles. **j** Summary of cycling lifespan of recently reported Li-ion full cells

**Fig. 4:**
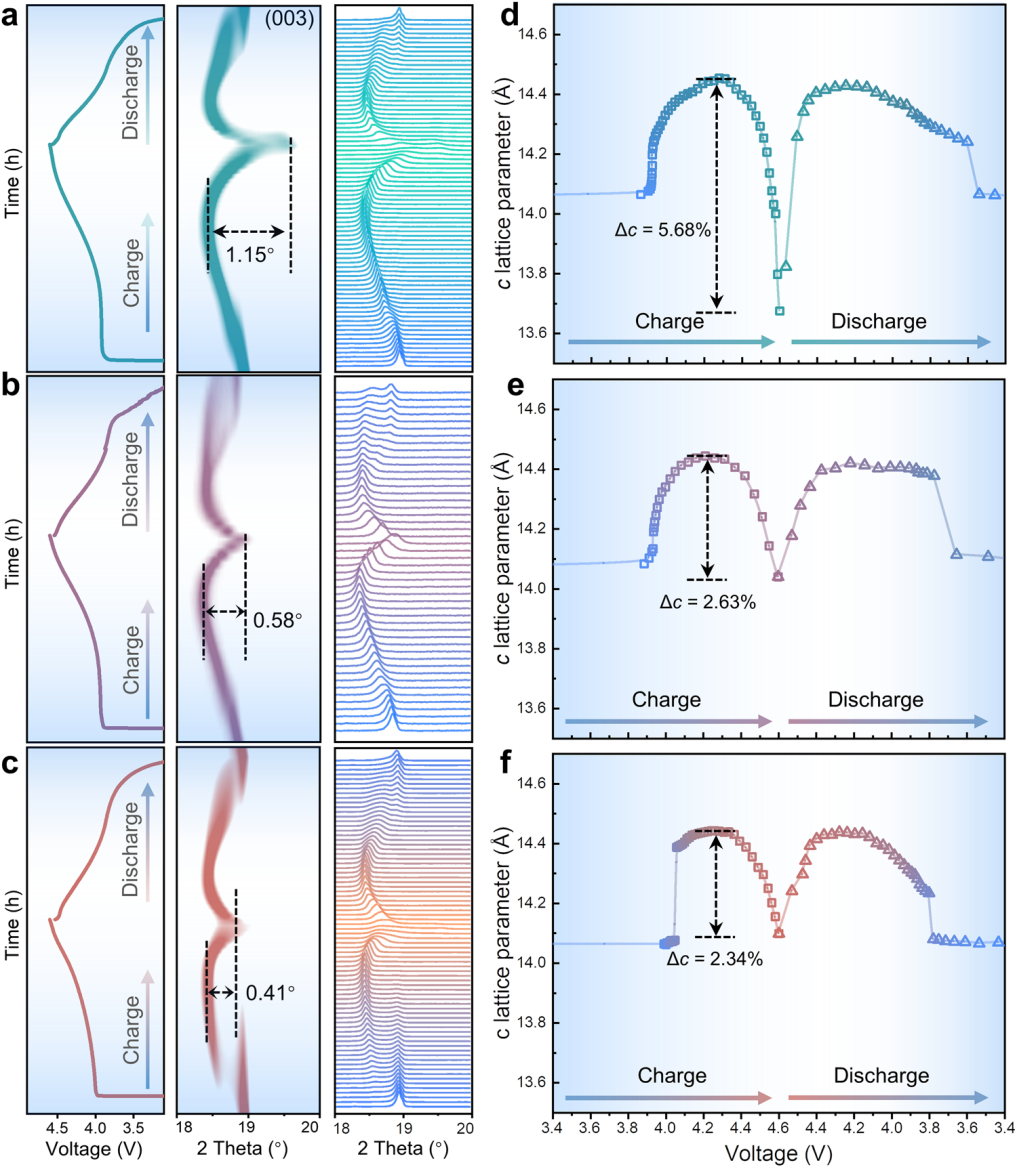
Structural evolutions of LCOs during initial charge/discharge cycle at 0.3C. Charge/discharge curves and *in-situ* XRD results of **a** LCO, **b** MAT-LCO and **c** CS-LCO for the initial cycle. The *c* lattice evolution of **d** LCO, **e** MAT-LCO and **f** CS-LCO as a function of charge/discharge voltage, fitted from the in situ XRD patterns. Δ*c* represents the variation of* c* lattice parameter from O3 to H1-3 phase transition during Li^+^ de-intercalation

The role played by the core–shell structure in stabilizing LCO cathode is evaluated by galvanostatic charge–discharge (GCD) profiles in half-cells within a voltage range of 3.0–4.6 V (vs. Li/Li^+^). As shown in Fig. S6, at an initial cycle of 0.1C, CS-LCO (208.6 mAh g^−1^) and MAT-LCO (210.5 mAh g^−1^) exhibit a lower specific capacity than LCO (211.4 mAh g^−1^) due to the co-doping of electrochemically inactive cations, which slightly sacrifices a small amount of capacity. However, the cycling stability of these three materials show obvious distinction. Figure 3a-c shows the continuous charge/discharge profiles from the 1st to the 150th cycle at 1C. The LCO displays a rapid capacity decay, resulting in a capacity of only 38.1 mAh g^−1^ after 150 cycles. In contrast, co-doping strategy is effective in improving stability, MAT-LCO exhibits a capacity of 120 mAh g^−1^. Notably, the capacity of CS-LCO continues to increase, reaching 185.2 mAh g^−1^. Figure 3d-f shows the differential capacity (dQ/dV) curves measured at 5th, 75th and 150th cycles for those cathodes, respectively. For the first few cycles, all cathodes exhibit the characteristic peaks at ~ 3.95, 4.21, and 4.55 V in the charge/discharge profiles. For LCO, an increased polarization and weakened characteristic peak can be observed after 150th cycles, indicating its poor structural stability under the high upper cut-off voltage of 4.6 V. Although MAT-LCO shows a little shift of the characteristic peak position, there is still a large drop in peak intensity. In contrast, the characteristic peaks of CS-LCO keep stable, indicating enhanced structural reversibility of cathode promoted by the cation co-doping strategy. To further investigate the evolutions of voltage profiles, these charge/discharge curves are normalized in Fig. S7. The electrochemical profiles of CS-LCO stably retain their overall shape within 150 cycles, whereas LCO suffers from dramatic voltage decay, losing its original profile. In addition, the average discharge voltages of LCO decreases from 3.901 to 3.503 V, MAT-LCO decreases from 3.874 to 3.781 V, while that of CS-LCO slightly increases from 3.882 to 3.925 V (Fig. S6d). We believe that the gradually increased average voltage and reversible capacity of CS-LCO can be ascribed to the progressive depolarization upon repeated charge/discharge cycles. The high roll strength makes low electrode porosity and inadequate infiltration for all LCO samples, indicating somewhat large electrochemical polarization. The LCO with large volume changes (Fig. 4a) and MAT-LCO with high specific surface area will result in the full infiltration of electrolyte and reduction of polarization within 10 cycles. While the weak lattice volume changes of CS-LCO (Fig. 4c) make the gradual contact between the CS-LCO and electrolyte, corresponding to the increasing average voltage and reversible capacity within 150 cycles. Even after 300 charge/discharge cycles, CS-LCO still exhibits a good capacity retention of 88.7% under 4.6 V, whereas that of LCO almost reaches zero within the same number of cycles (Fig. 3g). The rate performance (Fig. 3h) shows that the CS-LCO delivers a large capacity of 163.8 mAh g^−1^ at a high rate of 5C, much higher than those of LCO (40 mAh g^−1^) and MAT-LCO (125 mAh g^−1^), suggesting its outstanding high-rate property. The CS-LCO electrode also shows reduced electrochemical polarization as the rates exceed 2C (Fig. S8). Moreover, the cycling stability under higher rate of 5C and higher upper cut-off voltage of 4.7 V are measured (Figs. S9-S10). The CS-LCO shows a capacity retention of 90.9% (157 mAh g^−1^) under 4.6 V after 300 cycles at 5C. And it also shows a capacity retention of 61.7% (121 mAh g^−1^) after 300 cycles even under the higher cut-off voltage of 4.7 V. Besides, the CS-LCO//graphite full-cells exhibit exceptional long-term cycling stability, as evidenced by the capacity retention of 84.68% (569.9 Wh kg^−1^) after 300 cycles at 1C (Fig. 3i). The comparison of reported Li-ion full cells with this work is exhibited in Fig. 3j [19, 38–43], where the CS-LCO//graphite full cell reported in this work shows superior long-term cycling stability. Based on these observations, we can conclude that the core–shell structure synthesized by the co-doping strategy is effective to maintain the stability of LCO and reduce its voltage drop.

To gain deeper insights into the structural evolutions of cathode under high voltage, *in-situ* XRD is conducted to investigate the variations in lattice parameters and phase transition behavior of LCO, MAT-LCO and CS-LCO cathodes. Figure 4a-c shows the stacked peak profiles along with the first change-discharge cycle at 0.3C. As shown in Fig. 4d-f, during charge from 3.0 to 4.2 V, phase transition from the H1 phase to the M1 phase occurs [8]. The evolution of the *c* lattice parameter, which is calculated from the (003) peak, shows similar changes for all three samples. The lattice parameter ranges from 14.06 to 14.43 Å. Upon charging to 4.6 V, distinct differences in the *c* lattice evolution are observed. LCO (from 14.43 to 13.67 Å, Δ*c* = 5.68%) exhibits a significant contraction, whereas MAT-LCO (from 14.44 to 14.04 Å, Δ*c* = 2.63%) and CS-LCO (from 14.42 to 14.09 Å, Δ*c* = 2.34%) display a similar and slight contraction. Those findings suggest that pristine LCO suffers from a larger volume change (O3 to H1-3) under high voltage. As the structure transforms from O3 to H1-3, shift of the O–Co–O slabs occurs (along with lithium rearrangement) and the *c*-lattice of the unit cell shrinks dramatically, resulting in internal stress accumulation and structure damage [7, 44]. In contrast, MAT-LCO and CS-LCO effectively suppress undesired lattice distortion, indicating that the layered structure is well preserved even in the highly delithiated state. Additionally, CS-LCO have a little bit higher voltage platform at the beginning of charge. As we discussed before, some Co^3+^ ions are replaced by Ti^4+^ ions, which may lead to the extraction of some Li^+^ ions for charge balance, resulting in a higher voltage platform during the charge process.

To further explore the changes of interfacial impedance under varied state of charge (SOC), in situ electrochemical impedance spectroscopy (EIS) experiments are performed in the initial cycle (Fig. S11). Table S1 shows the fitted electrochemical parameters based on the equivalent circuit shown in Fig. S12, in which *R*_*sf*_ and *R*_*ct*_ refer to the resistances of surface film and charge transfer, respectively. The *R*_*sf*_ and *R*_*ct*_ decrease gradually as the charging voltage increases from 3.2 to 4.6 V. In the subsequent discharge process, the *R*_*sf*_ and *R*_*ct*_ both increase continuously from 4.6 to 3.0 V. The CS-LCO exhibits lower electrochemical impedance than that of LCO in the initial charge/discharge process, indicating decreased charge transfer resistance and enhanced Li^+^ ion diffusion during redox reactions. In addition, as shown in Fig. S13, the polarization becomes more significant as the cut-off voltage increases, which is attributed to structural decay and electrolyte decomposition at higher voltages.

**Fig. 5:**
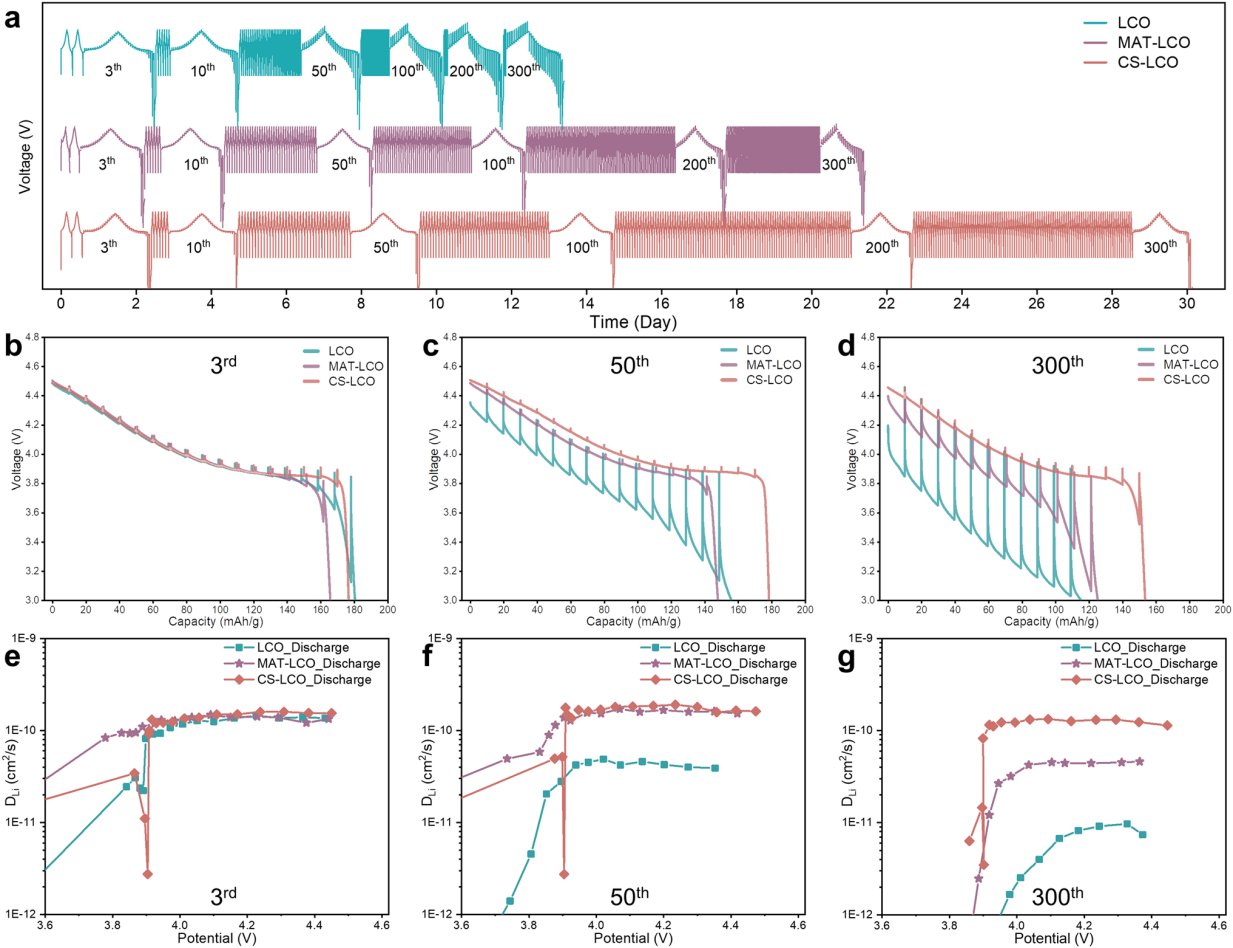
Li^+^ ion diffusion. **a** Cycling performance and overpotential properties of LCO, MAT-LCO and CS-LCO. The GITT curves of **b** LCO, **c** MAT-LCO and **d** CS-LCO at the 3rd, 50th, and 300th cycle. The corresponding Li^+^ ion diffusion coefficients of **e** LCO, **f** MAT-LCO and **g** CS-LCO at the 3rd, 50th, and 300th cycle

The galvanostatic intermittent titration technique (GITT) is further used to quantitatively evaluate the chemical diffusion coefficient of Li^+^ ion (*D*_*Li*+_, cm^2^ s^−1^) by different methods [45–47]. GITT measurements are conducted at 0.3C in the initial two cycle and 1C in 3rd–300th cycles. As shown in Fig. 5a-d, CS-LCO exhibits the smallest electrode polarization during the 3rd–300th cycle, whereas LCO and MAT-LCO exhibit rapid increase of electrode polarization since the 50th cycle. Besides, CS-LCO takes 30 days to complete 300 cycles, which is longer than LCO (13 days) and MAT-LCO (21 days). The longer cycling time means CS-LCO has the better cycling stability. The typical potential versus time profiles of LCO, MAT-LCO and CS-LCO are shown in Fig. S14. A linear relationship between potential and τ^1/2^ can be observed, and *D*_*Li*+_ can be calculated based on the Fick’s second law as follows [48, 49]:

1 $$D=\frac{4}{\pi \tau }{(\frac{m{V}_{M}}{MA})}^{2}{(\frac{\Delta {E}_{S}}{\Delta {E}_{\tau }})}^{2}$$

where *m* and *M* indicate the mass and molar mass of the electrode material, respectively. *V*_*M*_ (cm^3^ mol^−1^) refers to their molar volume, and *A* (cm^2^) stands for their active area. The* D*_*Li*+_ evolution of cathodes during cycling is shown in Fig. 5e-g. The calculated *D*_*Li*+_ values are similar for the three cathodes during the 3^rd^ cycle, where the average *D*_*Li*+_ is around 1.4 × 10^−10^ cm^2^ s^−1^. The average *D*_*Li*+_ of CS-LCO has almost no change during the 50th–300th cycle, while that of LCO drastically decreases. The GITT results confirm that the effectively co-doping strategy can maintain the stable Li^+^ diffusivity. As confirmed by *in-situ* XRD, LCO undergoes a large contraction of ***c*** lattice due to the phase transition of O3 to H1-3 when charging voltage exceeds 4.5 V. This large contraction result in severe structural distortion and the formation of a thick CEI during repeated cycles, which potentially block the Li^+^ diffusion [18, 25]. In contrast, cation co-doping in CS-LCO helps facilitate the (de)intercalation of Li^+^ ions and prevent the structure distortion, hence maintaining a high Li^+^ diffusion coefficient even after long-term cycling.

To further examine the change of surface composition of cathode after cycling, XPS was introduced to characterize the cathode/electrolyte interphase (CEI) layer. In general, C = O/Li_2_CO_3_ (531.0 eV) and CO_3_^2−^ (290.0 eV) are related to the presence of lithium carbonate. As shown in Fig. S15, it can be found that, compared with LCO, the amount of carbonate was obviously reduced after co-doping (MAT-LCO and CS-LCO). The presence of more carbonate on the surface always results in higher impedance, which is consistent with EIS results as shown in Fig. S16. As for F 1*s*, the peaks are primarily assigned to LiF (685 eV) and PVdF (687 eV). LiF is the main F-containing components in CEI due to the prevailing HF attack at cathode surface, resulting in capacity fading under high voltage [50]. The relatively low content of LiF in CS-LCO indicates a thinner CEI layer, and the reduced side reaction at cathode/electrolyte interface. As for the O 1* s*, the higher intensity of lattice oxygen (529.5 eV) obtained from CS-LCO confirms the suppressed lattice oxygen oxidizing ability.

Previous studies reported the origin and evolution of gliding layers and microcracks under high voltages [51]. Charging to a high voltage, more Li^+^ ions are extracted from the original structure, resulting in the slab gliding of layered structure. Typically, these gliding traces will disappear in the subsequent discharge process if the structural change is reversible. However, there are also irreversible gliding events that accumulate over long-term cycling and form microcracks, exposing fresh surfaces to the electrolyte. The penetration of the liquid electrolyte along the layered gliding increases the risk of electrolyte decomposition.

**Fig. 6:**
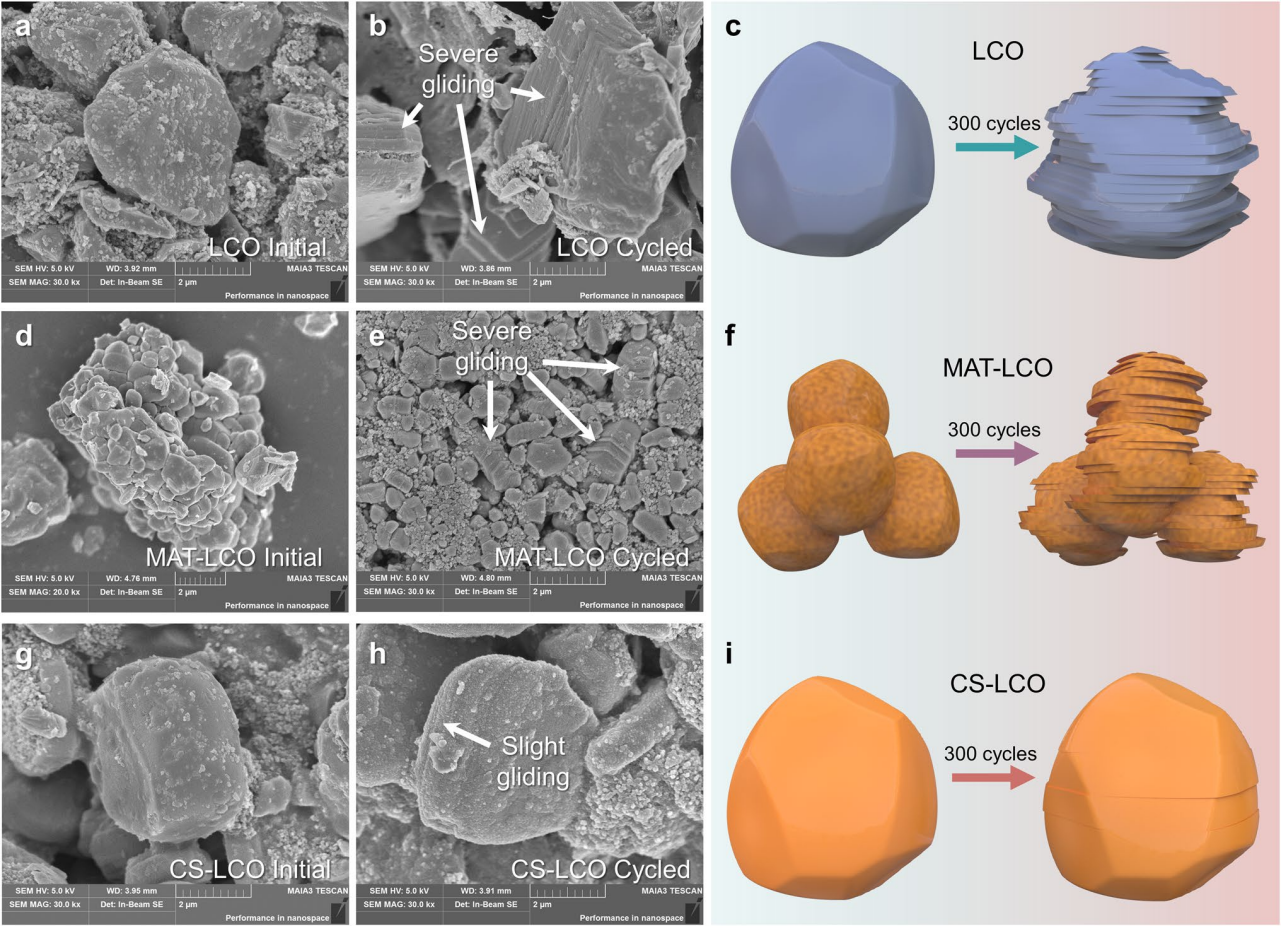
Structural evolutions after long-term cycling. SEM images and the corresponding schematic diagrams of the initial and after 300 cycles of **a-c** LCO, **d-f** MAT-LCO and **d-i** CS-LCO cathodes

Figure 6 shows the observation of lattice plane gliding at 4.6 V after 300 cycles. For LCO, the gliding traces are evident as in-plane shear along the (003) plane, vertical to the *c* axis of the layered structure, which ultimately results in the accumulation of irreversible structural deformation and the creation of significant cracks. Moreover, serve gliding is also observed in small primary particles of MAT-LCO, possibly due to the larger contact area with the electrolyte. In contrast, the entire structure of CS-LCO is well maintained after 300 cycles, showing almost reversible gliding. Notably, due to the capacity decay, LCO can only be cycled for 195 h at 1C (relative to the theoretical capacity), whereas CS-LCO can be cycled for 825 h, four times the cycling time of LCO (Fig. 5a). These results indicate the crucial role played by co-doping induced core–shell structure in effective suppressing irreversible gilding and maintaining structural integrity.

As shown in Figs. 4 and 6, LCO undergoes a significant phase transition process under 4.6 V. The accumulation of volume changes and layer gilding eventually leads to the collapse of the structure. To further illustrate the structural degradation after long-term cycling, *ex-situ* XRD is performed. The (003) peak of LCO and MAT-LCO moves to a lower diffraction angle after 100 cycles (Fig. S17), indicating the Li^+^ irreversible de-intercalation probably caused by structural transition and accumulation of CEI film during continuous cycling. In contrast, CS-LCO exhibits a weak shift of (003) peak and enhanced structural reversibility. In addition, the collapse of layered structure and the larger surface area can increase the possibility of the liquid electrolyte decomposition during long-term cycling. This could result in the larger charge transfer impedance, as shown in Fig. S16. The redox kinetics are explored through cyclic voltammetry (CV) scanning as shown in Fig. S18. During the discharge process, the cathodic peaks at 4.45, 4.1 and 3.8 V relate to the phase transition of H1-3/O3, the order–disorder phase transition, and the phase transition of M2/H3, respectively [41, 52]. With increasing scanning cycles, the peaks of LCO are weakened gradually and the polarization phenomena become more pronounced. These observations suggest that the phase transitions of H1-3/O3 and H2/H1 become irreversible. Notably, the peaks of the anodic process of CS-LCO are sharper than LCO, indicating faster charge transfer kinetics. After 100 cycles, the redox peak positions and intensities of CS-LCO remain almost unchanged, demonstrating its exceptional structural reversibility and stability during long-term cycling at high voltages.

## Conclusions

In summary, a single-crystal core–shell LiCoO_2_ (CS-LCO) cathode is designed and synthesized by a simple two-step co-doping strategy. This reliable co-doping strategy results in the preservation of single-crystal morphology and the suppression of phase transition under deep delithiation. In this designed core–shell structure, high-diffusivity Mg^2+^/Al^3+^ ions are doped into the core region of single-crystal particles to optimize physical properties, such as conductivity and Li^+^ ion diffusivity, while low-diffusivity Ti^4+^ ions enrich the shell layer to enhance the surface structure stability. Under a high cut-off voltage of 4.6 V, CS-LCO exhibits a stable capacity of 159.8 mAh g^−1^ (88.73% retention) at 1C after 300 cycles, and reaches a high capacity of 157 mAh g^−1^ (90.9% retention) at 5C after 300 cycles. Even at a higher cut-off voltage of 4.7 V, CS-LCO still exhibits good retention of ~ 61.7% at 1C after 300 cycles. These achievements may provide guidance for the rational design of core–shell structured cathodes with multiple dopants involving low-diffusivity cations (Zr^4+^, Ta^5+^, W^6+^, etc.) and high-diffusivity ones (Zn^2+^, Ni^2+^, Fe^3+^, etc.).

## Supplementary Information

Below is the link to the electronic supplementary material.

Supplementary file1 (PDF 2202 kb)

## References

[1] J.B. Goodenough, Y. Kim, Challenges for rechargeable Li batteries. Chem. Mater. **22**(3), 587–603 (2010). 10.1021/cm901452z

[2] K. Zhao, Y. Wang, L. Han, Y. Wang, X. Luo et al., Nanogenerator-based self-charging energy storage devices. Nano-Micro Lett. **11**(1), 19 (2019). 10.1007/s40820-019-0251-7 10.1007/s40820-019-0251-7 PMC7770842 34137958

[3] J. Xu, Critical review on cathode–electrolyte interphase toward high-voltage cathodes for Li-Ion batteries. Nano-Micro Lett. **14**(1), 166 (2022). 10.1007/s40820-022-00917-2 10.1007/s40820-022-00917-2 PMC9381680 35974213

[4] Y. Liu, T. Zeng, G. Li, T. Wan, M. Li et al., The surface double-coupling on single-crystal LiNi_0.8_Co_0.1_Mn_0.1_O_2_ for inhibiting the formation of intragranular cracks and oxygen vacancies. Energy Stor. Mater. **52**, 534–546 (2022). 10.1016/j.ensm.2022.08.026

[5] H. Zhu, Y. Tang, K.M. Wiaderek, O.J. Borkiewicz, Y. Ren et al., Spontaneous strain buffer enables superior cycling stability in single-crystal nickel-rich NCM cathode. Nano Lett. **21**(23), 9997–10005 (2021). 10.1021/acs.nanolett.1c03613 34813330 10.1021/acs.nanolett.1c03613

[6] Z. Zhong, L. Chen, S. Huang, W. Shang, L. Kong et al., Single-crystal LiNi_0.5_Co_0.2_Mn_0.3_O_2_: a high thermal and cycling stable cathodes for lithium-ion batteries. J. Mater. Sci. **55**(7), 2913–2922 (2020). 10.1007/s10853-019-04133-z

[7] Z. Chen, J.R. Dahn, Methods to obtain excellent capacity retention in LiCoO_2_ cycled to 4.5 V. Electrochim. Acta **49**(7), 1079–1090 (2004). 10.1016/j.electacta.2003.10.019

[8] G.G. Amatucci, J.M. Tarascon, L.C. Klein, CoO_2_, the end member of the Li_x_CoO_2_ solid solution. J. Electrochem. Soc. **143**(3), 1114–1123 (1996). 10.1149/1.1836594

[9] Y. Lyu, X. Wu, K. Wang, Z. Feng, T. Cheng et al., An overview on the advances of LiCoO_2_ cathodes for lithium-ion batteries. Adv. Energy Mater. **11**(2), 2000982 (2021). 10.1002/aenm.202000982

[10] D. Takamatsu, Y. Koyama, Y. Orikasa, S. Mori, T. Nakatsutsumi et al., First in situ observation of the LiCoO_2_ electrode/electrolyte interface by total-reflection x-ray absorption spectroscopy. Angew. Chem. Int. Ed. **51**(46), 11597–11601 (2012). 10.1002/anie.201203910 10.1002/anie.201203910 23065948

[11] G.G. Amatucci, J.M. Tarascon, L.C. Klein, Cobalt dissolution in LiCoO_2_-based non-aqueous rechargeable batteries. Solid State Ionics **83**(1), 167–173 (1996). 10.1016/0167-2738(95)00231-6

[12] Q. Liu, X. Su, D. Lei, Y. Qin, J. Wen et al., Approaching the capacity limit of lithium cobalt oxide in lithium ion batteries via lanthanum and aluminium doping. Nat. Energy **3**(11), 936–943 (2018). 10.1038/s41560-018-0180-6

[13] W. Kong, J. Zhang, D. Wong, W. Yang, J. Yang et al., Tailoring Co3d and O2p band centers to inhibit oxygen escape for stable 4.6 V LiCoO_2_ cathodes. Angew. Chem. Int. Ed. **133**(52), 27308–27318 (2021). 10.1002/anie.202112508 10.1002/anie.202112508 34668282

[14] J. Ahn, J. Kang, M.K. Cho, H. Park, W. Ko et al., Selective anionic redox and suppressed structural disordering enabling high-energy and long-life Li-rich layered-oxide cathode. Adv. Energy Mater. **11**(47), 2102311 (2021). 10.1002/aenm.202102311

[15] M. Wang, X. Feng, H. Xiang, Y. Feng, C. Qin et al., A novel protective strategy on high-voltage LiCoO_2_ cathode for fast charging applications: Li_1.6_Mg_1.6_Sn_2.8_O_8_ double layer structure via SnO_2_ surface modification. Small Methods **3**(11), 1900355 (2019). 10.1002/smtd.201900355

[16] Y. Huang, Y. Zhu, H. Fu, M. Ou, C. Hu et al., Mg-pillared LiCoO_2_: towards stable cycling at 4.6 V. Angew. Chem. Int. Ed. **60**(9), 4682–4688 (2021). 10.1002/anie.202014226 10.1002/anie.202014226 33191621

[17] A. Liu, J. Li, R. Shunmugasundaram, J.R. Dahn, Synthesis of Mg and Mn doped LiCoO_2_ and effects on high voltage cycling. J. Electrochem. Soc. **164**(7), A1655–A1664 (2017). 10.1149/2.1381707jes

[18] L. Wang, J. Ma, C. Wang, X. Yu, R. Liu et al., A novel bifunctional self-stabilized strategy enabling 4.6 V LiCoO2 with excellent long-term cyclability and high-rate capability. Adv. Sci. **6**(12), 1900355 (2019). 10.1002/advs.201900355 10.1002/advs.201900355 PMC6662074 31380171

[19] J.N. Zhang, Q. Li, C. Ouyang, X. Yu, M. Ge et al., Trace doping of multiple elements enables stable battery cycling of LiCoO_2_ at 4.6 V. Nat. Energy **4**(7), 594–603 (2019). 10.1038/s41560-019-0409-z

[20] Y. Hong, X. Huang, C. Wei, J. Wang, J. Zhang et al., Hierarchical defect engineering for LiCoO_2_ through low-solubility trace element doping. Chem **6**(10), 2759–2769 (2020). 10.1016/j.chempr.2020.07.017

[21] M. Hirooka, T. Sekiya, Y. Omomo, M. Yamada, H. Katayama et al., Improvement of float charge durability for LiCoO_2_ electrodes under high voltage and storage temperature by suppressing O1-Phase transition. J. Power. Sources **463**, 228127 (2020). 10.1016/j.jpowsour.2020.228127

[22] H. Ronduda, M. Zybert, A. Szczęsna, T. Trzeciak, A. Ostrowski et al., Addition of yttrium oxide as an effective way to enhance the cycling stability of LiCoO_2_ cathode material for Li-ion batteries. Solid State Ionics **355**, 115426 (2020). 10.1016/j.ssi.2020.115426

[23] J. Xiang, Y. Wei, Y. Zhong, Y. Yang, H. Cheng et al., Building practical high-voltage cathode materials for lithium-ion batteries. Adv. Mater. **34**(52), 2200912 (2022). 10.1002/adma.202200912 10.1002/adma.202200912 35332962

[24] J. Langdon, A. Manthiram, A perspective on single-crystal layered oxide cathodes for lithium-ion batteries. Energy Stor. Mater. **37**, 143–160 (2021). 10.1016/j.ensm.2021.02.003

[25] C. Yang, X. Liao, X. Zhou, C. Sun, R. Qu et al., Phosphate-rich interface for a highly stable and safe 4.6 V LiCoO_2_ cathode. Adv. Mater. **35**(14), 2210966 (2023). 10.1002/adma.202210966 10.1002/adma.202210966 36649735

[26] S. Kim, S. Choi, K. Lee, G.J. Yang, S.S. Lee et al., Self-assembly of core–shell structures driven by low doping limit of Ti in LiCoO_2_: first-principles thermodynamic and experimental investigation. Phys. Chem. Chem. Phys. **19**(5), 4104–4113 (2017). 10.1039/C6CP08114B 28111667 10.1039/c6cp08114b

[27] S. Song, Y. Li, K. Yang, Z. Chen, J. Liu et al., Interplay between multiple doping elements in high-voltage LiCoO_2_. J. Mater. Chem. A **9**(9), 5702–5710 (2021). 10.1039/D0TA09931G

[28] G. Kresse, J. Furthmüller, Efficient iterative schemes for ab initio total-energy calculations using a plane-wave basis set. Phys. Rev. B **54**(16), 11169–11186 (1996). 10.1103/PhysRevB.54.11169 10.1103/physrevb.54.11169 9984901

[29] G. Kresse, D. Joubert, From ultrasoft pseudopotentials to the projector augmented-wave method. Phys. Rev. B **59**(3), 1758–1775 (1999). 10.1103/PhysRevB.59.1758

[30] S.L. Dudarev, G.A. Botton, S.Y. Savrasov, C.J. Humphreys, A.P. Sutton, Electron-energy-loss spectra and the structural stability of nickel oxide: An LSDA+U study. Phys. Rev. B **57**(3), 1505–1509 (1998). 10.1103/PhysRevB.57.1505

[31] J.P. Perdew, A. Ruzsinszky, G.I. Csonka, O.A. Vydrov, G.E. Scuseria et al., Restoring the density-gradient expansion for exchange in solids and surfaces. Phys. Rev. Lett. **100**(13), 136406 (2008). 10.1103/PhysRevLett.100.136406 18517979 10.1103/PhysRevLett.100.136406

[32] J.P. Perdew, K. Burke, M. Ernzerhof, Generalized gradient approximation made simple. Phys. Rev. Lett. **77**(18), 3865 (1996). 10.1103/PhysRevLett.77.3865 10062328 10.1103/PhysRevLett.77.3865

[33] A. Jain, S.P. Ong, G. Hautier, W. Chen, W.D. Richards et al., Commentary: The materials project: a materials genome approach to accelerating materials innovation. APL Mater. **1**(1), 011002 (2013). 10.1063/1.4812323

[34] G. Henkelman, B.P. Uberuaga, H. Jónsson, A climbing image nudged elastic band method for finding saddle points and minimum energy paths. J. Chem. Phys. **113**(22), 9901–9904 (2000). 10.1063/1.1329672

[35] M. Cai, Y. Dong, M. Xie, W. Dong, C. Dong et al., Stalling oxygen evolution in high-voltage cathodes by lanthurization. Nat. Energy **8**(2), 159–168 (2023). 10.1038/s41560-022-01179-3

[36] C.-W. Wang, Y. Zhou, J.-H. You, J.-D. Chen, Z. Zhang et al., High-voltage LiCoO_2_ material encapsulated in a Li_4_Ti_5_O_12_ ultrathin layer by high-speed solid-phase coating process. ACS Appl. Energy Mater. **3**(3), 2593–2603 (2020). 10.1021/acsaem.9b02291

[37] Y. Wang, Q. Zhang, Z.C. Xue, L. Yang, J. Wang et al., An in situ formed surface coating layer enabling LiCoO_2_ with stable 4.6 V high-voltage cycle performances. Adv. Energy Mater. **10**(28), 2001413 (2020). 10.1002/aenm.202001413

[38] Z. Li, A. Li, H. Zhang, F. Ning, W. Li et al., Multi-scale stabilization of high-voltage LiCoO_2_ enabled by nanoscale solid electrolyte coating. Energy Stor. Mater. **29**, 71–77 (2020). 10.1016/j.ensm.2020.03.031

[39] J.H. Shim, J.M. Han, J.H. Lee, S. Lee, Mixed electronic and ionic conductor-coated cathode material for high-voltage lithium ion battery. ACS Appl. Mater. Interfaces **8**(19), 12205–12210 (2016). 10.1021/acsami.6b03113 27127906 10.1021/acsami.6b03113

[40] Z. Zhu, H. Wang, Y. Li, R. Gao, X. Xiao et al., A surface Se-substituted LiCo[O_2−δ_Se_δ_] cathode with ultrastable high-voltage cycling in pouch full-cells. Adv. Mater. **32**(50), 2005182 (2020). 10.1002/adma.202005182 10.1002/adma.202005182 33174311

[41] J. Qian, L. Liu, J. Yang, S. Li, X. Wang et al., Electrochemical surface passivation of LiCoO_2_ particles at ultrahigh voltage and its applications in lithium-based batteries. Nat. Commun. **9**(1), 4918 (2018). 10.1038/s41467-018-07296-6 30464176 10.1038/s41467-018-07296-6 PMC6249257

[42] Z. Feng, R. Rajagopalan, D. Sun, Y. Tang, H. Wang, In-situ formation of hybrid Li_3_PO_4_-AlPO_4_-Al(PO_3_)_3_ coating layer on LiNi_0.8_Co_0.1_Mn_0.1_O_2_ cathode with enhanced electrochemical properties for lithium-ion battery. Chem. Eng. J. **382**, 122959 (2020). 10.1016/j.cej.2019.122959

[43] X. Yang, C. Wang, P. Yan, T. Jiao, J. Hao et al., Pushing lithium cobalt oxides to 4.7 V by lattice-matched interfacial engineering. Adv. Energy Mater. **12**(23), 2200197 (2022). 10.1002/aenm.202200197

[44] W. Zheng, Q. Liu, Z. Wang, Z. Wu, S. Gu et al., Oxygen redox activity with small voltage hysteresis in Na_0.67_Cu_0.28_Mn_0.72_O_2_ for sodium-ion batteries. Energy Stor. Mater. **28**, 300–306 (2020). 10.1016/j.ensm.2020.03.016

[45] Y. Lai, H. Xie, P. Li, B. Li, A. Zhao et al., Ion-migration mechanism: an overall understanding of anionic redox activity in metal oxide cathodes of Li/Na-ion batteries. Adv. Mater. **34**(47), 2206039 (2022). 10.1002/adma.202206039 10.1002/adma.202206039 36165216

[46] H. Wang, Z. Cui, S.-A. He, J. Zhu, W. Luo et al., Construction of ultrathin layered MXene-TiN heterostructure enabling favorable catalytic ability for high-areal-capacity lithium–sulfur batteries. Nano-Micro Lett. **14**(1), 189 (2022). 10.1007/s40820-022-00935-0 10.1007/s40820-022-00935-0 PMC9482562 36114888

[47] Q. Ding, W. Zheng, A. Zhao, Y. Zhao, K. Chen et al., W-doping induced efficient tunnel-to-layered structure transformation of Na_0.44_Mn_1-x_W_x_O_2_: phase evolution, sodium-storage properties, and moisture stability. Adv. Energy Mater. **13**(21), 2203802 (2023). 10.1002/aenm.202203802

[48] H.-G. Jung, J. Hassoun, J.-B. Park, Y.-K. Sun, B. Scrosati, An improved high-performance lithium–air battery. Nat. Chem. **4**(7), 579–585 (2012). 10.1038/nchem.1376 22717445 10.1038/nchem.1376

[49] F. Li, K. Fan, Y. Tian, P. Hou, H. Zhang et al., General flux-free synthesis of single crystal Ni-rich layered cathodes by employing a Li-containing spinel transition phase for lithium-ion batteries. J. Mater. Chem. A **10**(31), 16420–16429 (2022). 10.1039/D2TA02865D

[50] Y.C. Lu, A.N. Mansour, N. Yabuuchi, Y. Shao-Horn, Probing the origin of enhanced stability of “AlPO_4_” nanoparticle coated LiCoO_2_ during cycling to high voltages: combined XRD and XPS studies. Chem. Mater. **21**(19), 4408–4424 (2009). 10.1021/cm900862v

[51] Y. Bi, J. Tao, Y. Wu, L. Li, Y. Xu et al., Reversible planar gliding and microcracking in a single-crystalline Ni-rich cathode. Science **370**(6522), 1313–1317 (2020). 10.1126/science.abc3167 33303612 10.1126/science.abc3167

[52] L. Wang, B. Chen, J. Ma, G. Cui, L. Chen, Reviving lithium cobalt oxide-based lithium secondary batteries-toward a higher energy density. Chem. Soc. Rev. **47**(17), 6505–6602 (2018). 10.1039/C8CS00322J 29956705 10.1039/c8cs00322j

