# Supplementary material for: Mitigating Lattice Distortion of High-Voltage LiCoO2 via Core-Shell Structure Induced by Cationic Heterogeneous Co-Doping for Lithium-Ion Batteries
Source: Nanomicro Lett. 2023 Dec 11;16:48. doi: 10.1007/s40820-023-01269-1 (PMC10713914; doi:10.1007/s40820-023-01269-1)
Supplement: Supplementary file 1 — Supplementary file1 (PDF 2202 kb) [file 40820_2023_1269_MOESM1_ESM.pdf]

Supporting Information for

Mitigating Lattice Distortion of High-Voltage $LiCoO_2$ *via* Core-Shell Structure Induced by Cationic Heterogeneous Codoping for Lithium-Ion Batteries

Zezhou Lin[1], Ke Fan[1], Tiancheng Liu[1], Zhihang Xu[2], Gao Chen[1], Honglei Zhang[1], Hao Li[1], Xuyun Guo[2], Xi Zhang[3], Ye Zhu[2], Peiyu Hou[4, *] and Haitao Huang[1, *]

[1] Department of Applied Physics and Research Institute for Smart Energy, The Hong Kong Polytechnic University, Hong Kong, P. R. China

[2] Department of Applied Physics, The Hong Kong Polytechnic University, Hong Kong, P. R. China

[3] Institute of Nanosurface Science and Engineering, Guangdong Provincial Key Laboratory of Micro/Nano Optomechatronics Engineering, Shenzhen University, Shenzhen, P. R. China

[4] School of Physics and Technology, University of Jinan, Jinan, Shandong, P. R. China

[*] Corresponding authors. E-mail: sps_houpy@ujn.edu.cn (P. Hou) and aphhuang@polyu.edu.hk (H. Huang)

Supplementary Figures and Tables

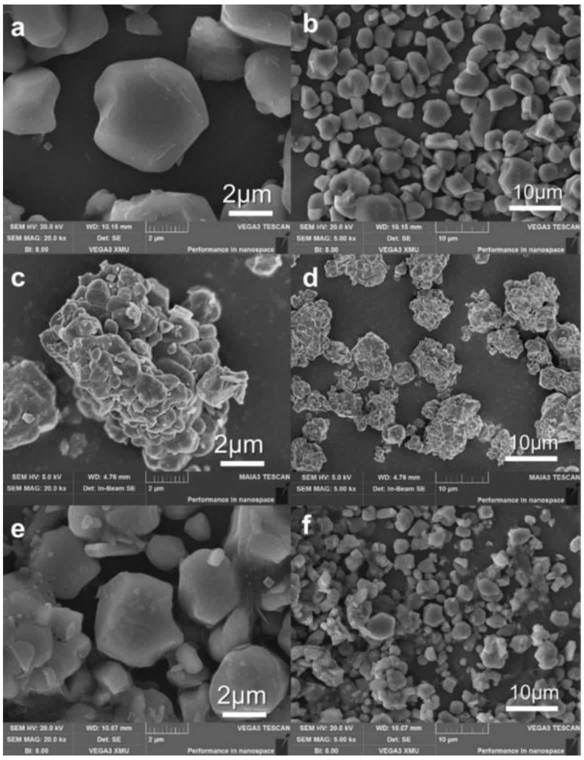

Fig. S1 SEM image of **a-b** LCO, **c-d** MAT-LCO, and **e-f** CS-LCO

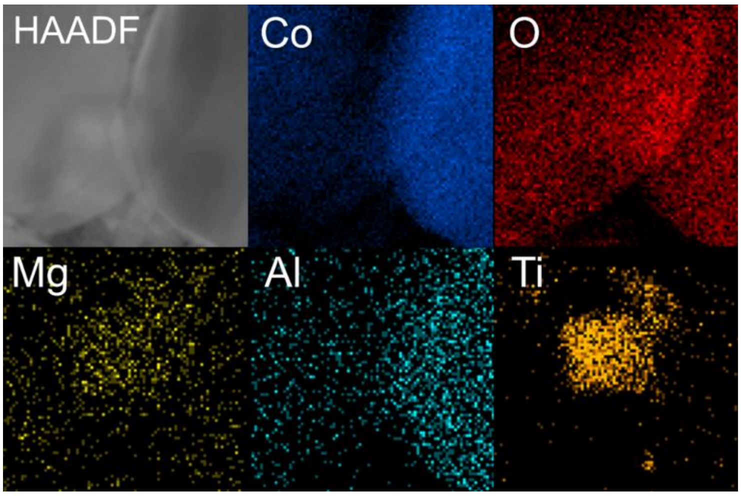

Fig. S2 EDS mapping of MAT-LCO

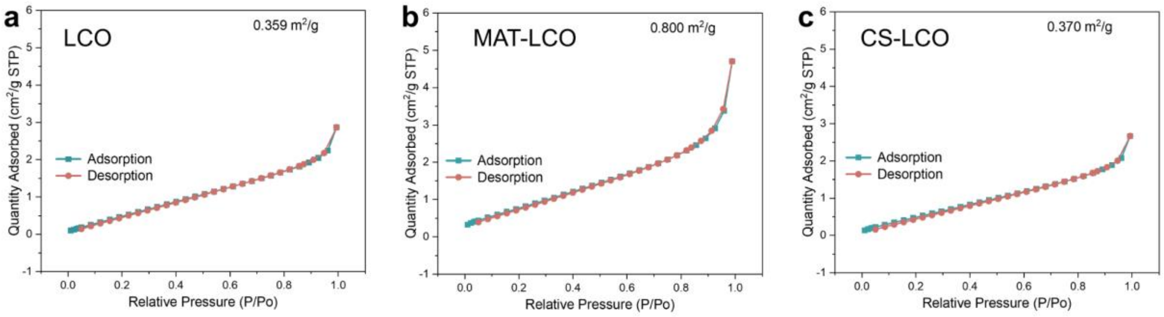

Fig. S3 BET diagram of **a** LCO, **b** MAT-LCO, and **c** CS-LCO

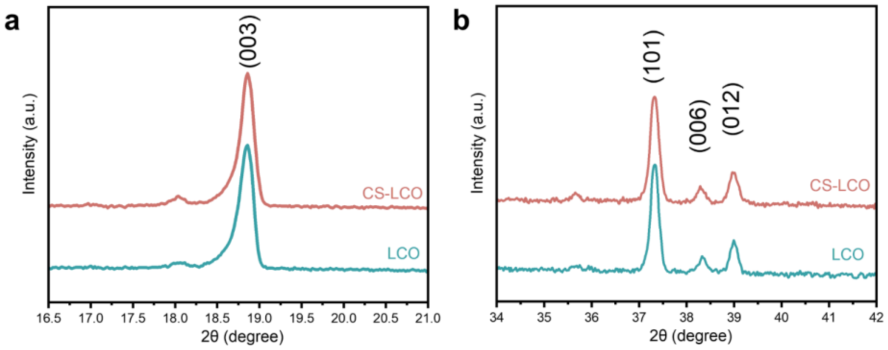

Fig. S4 a The (003) peak, and **b** (006) / (012) peak of LCO and CS-LCO

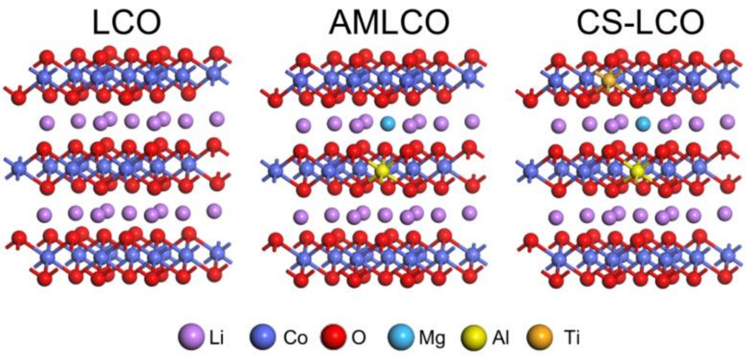

Fig. S5 Simplified structural models of LCO, AMLCO and CS-LCO

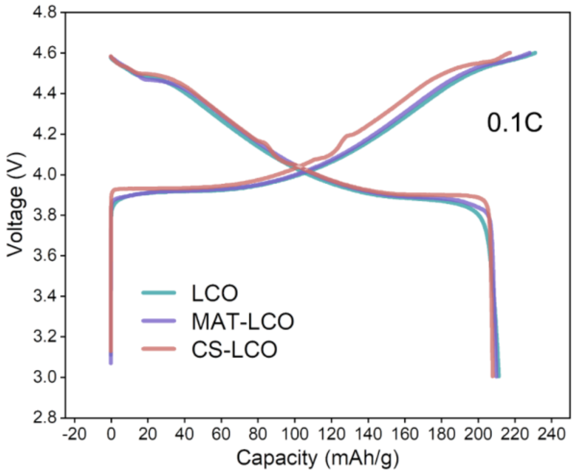

Fig. S6 Galvanostatic charge-discharge (GCD) profiles of LCO, MAT-LCO and CS-LCO during the first cycle (3.0-4.6V) at 0.1C

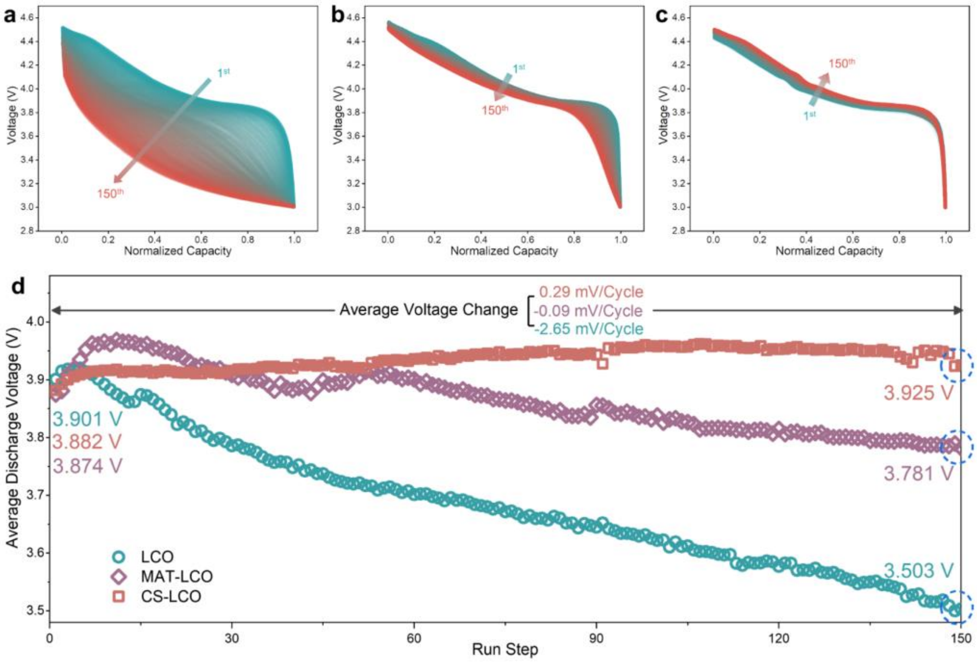

Fig. S7 Normalized charge/discharge profiles for 150 cycles of **a** LCO, **b** MAT-LCO and **c** CS-LCO. **d** Comparison of average discharge voltage at 1C under 4.6V from the 1st to the 150th cycles

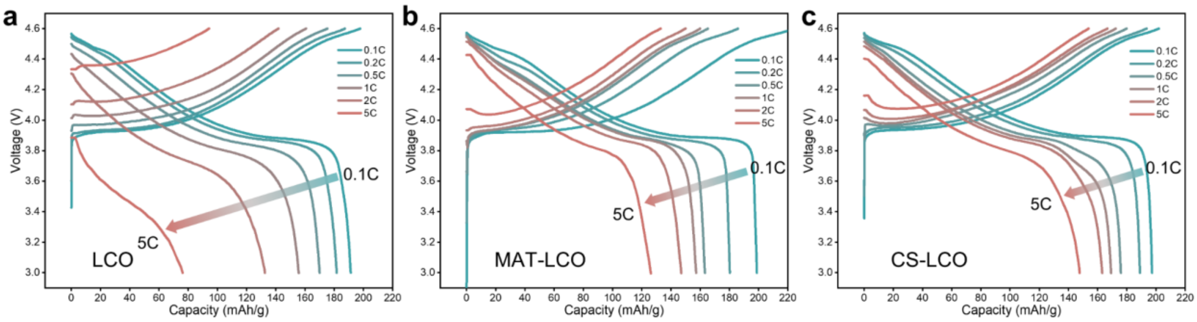

Fig. S8 Continuous charge/discharge curves at 3.0-4.6V from 0.1 C to 5C of **a** LCO, **b** MAT-LCO and **c** CS-LCO

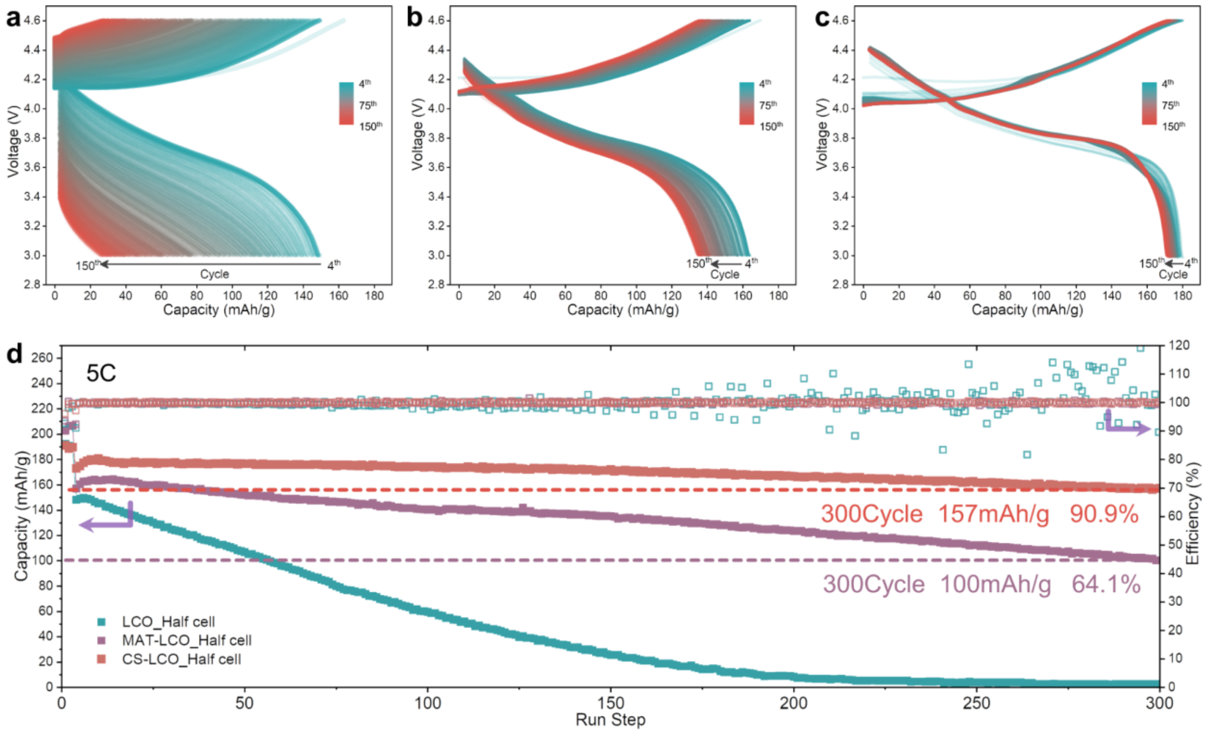

Fig. S9 High rate (5C) electrochemical performance. Continuous charge/discharge curves from the 4th to the 150th cycles of **a** LCO, **b** MAT-LCO and **c** CS-LCO under 4.6V. **d** Cycling stability of half-cells from the 1st to the 300th cycles at 4.6V, the 1st to 3rd cycles at 1C, the 4th to 300th at 5C

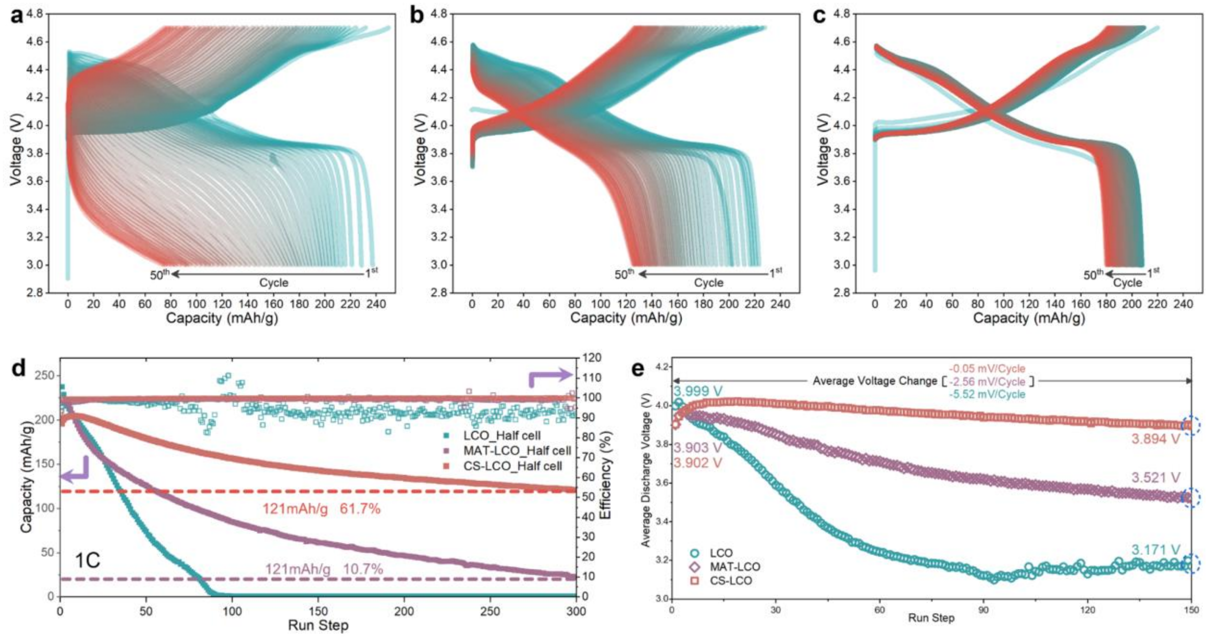

Fig. S10 High voltage (4.7V) electrochemical performance. Continuous charge/discharge curves from the 1st to the 50th cycles of **a** LCO, **b** MAT-LCO and **c** CS-LCO at 1C under 4.7V from 1st to 50th cycles. **d** Cycling stability of half-cell at 1C under 4.7V from 1st to 300th cycles. **e** Comparison of average discharge voltage at 1C under 4.7V from 1st to 150th cycles

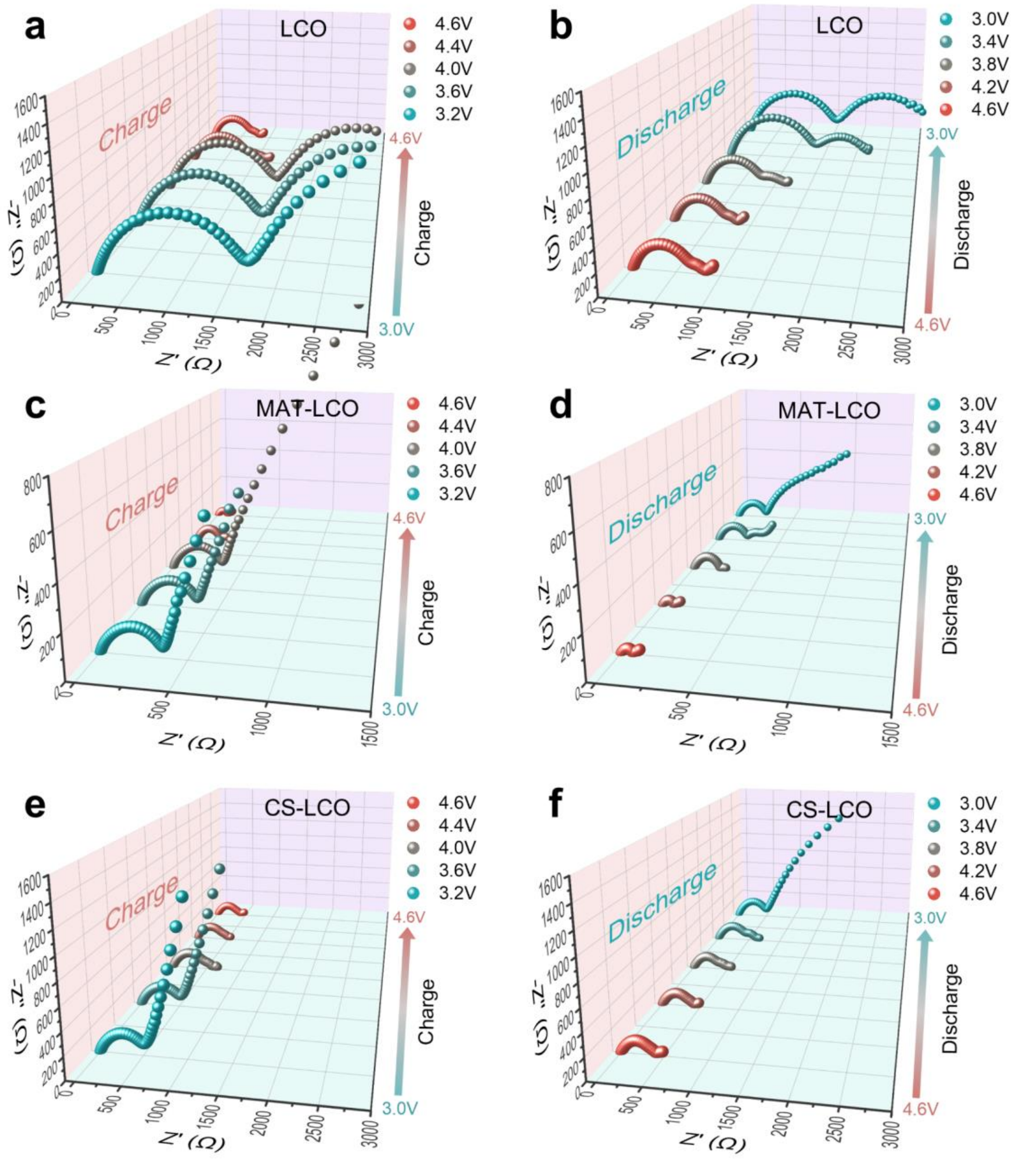

Fig. S11 Impedance evolution during the *in-situ* charge/discharge process for **a-b** LCO, **c-d** MAT-LCO and **e-f** CS-LCO in the range of 3.0-4.6 V at 0.1 C

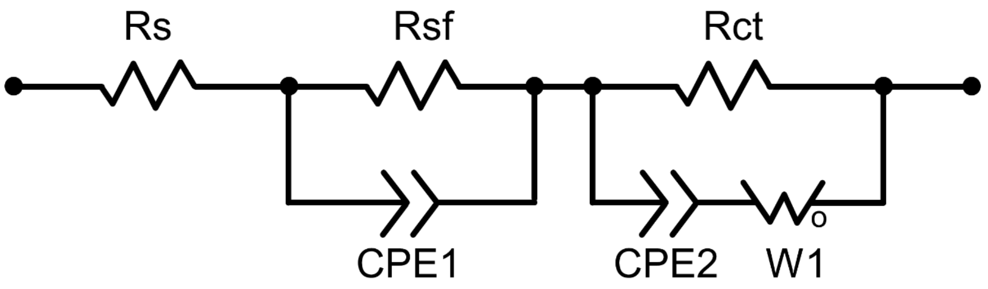

Fig. S12 An equivalent electrical circuit that describes the impedance behavior of LCO, MAT-LCO, and CS-LCO electrodes

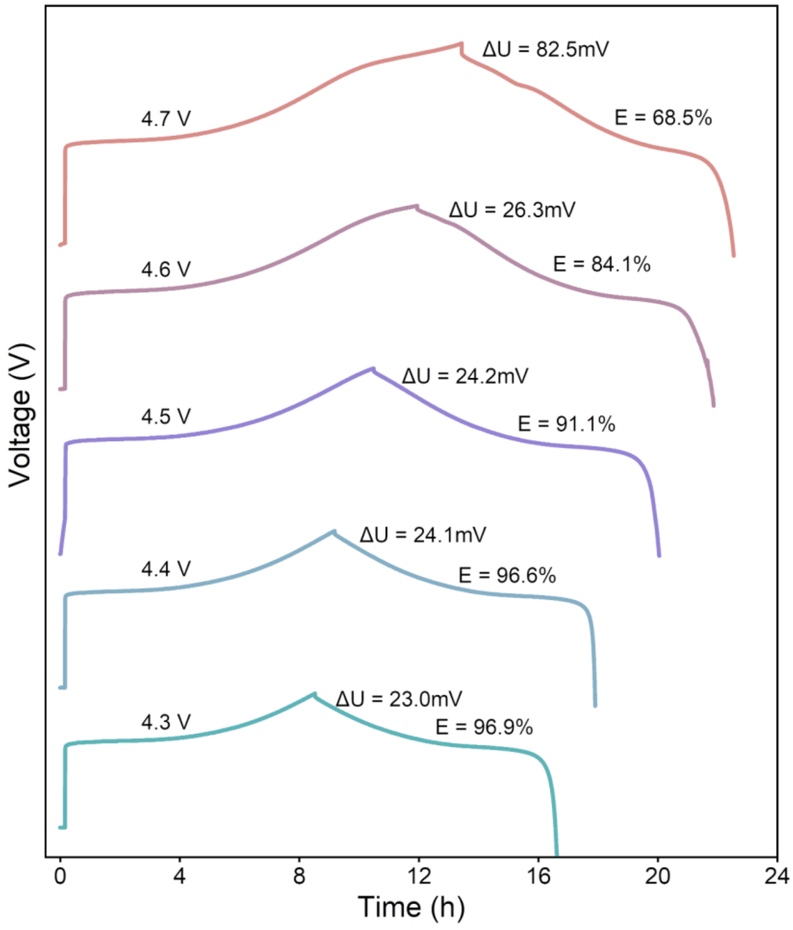

Fig. S13 The initial charge/discharge cycle of LCO under 4.3V - 4.7V. E indicate the coulombic efficiency

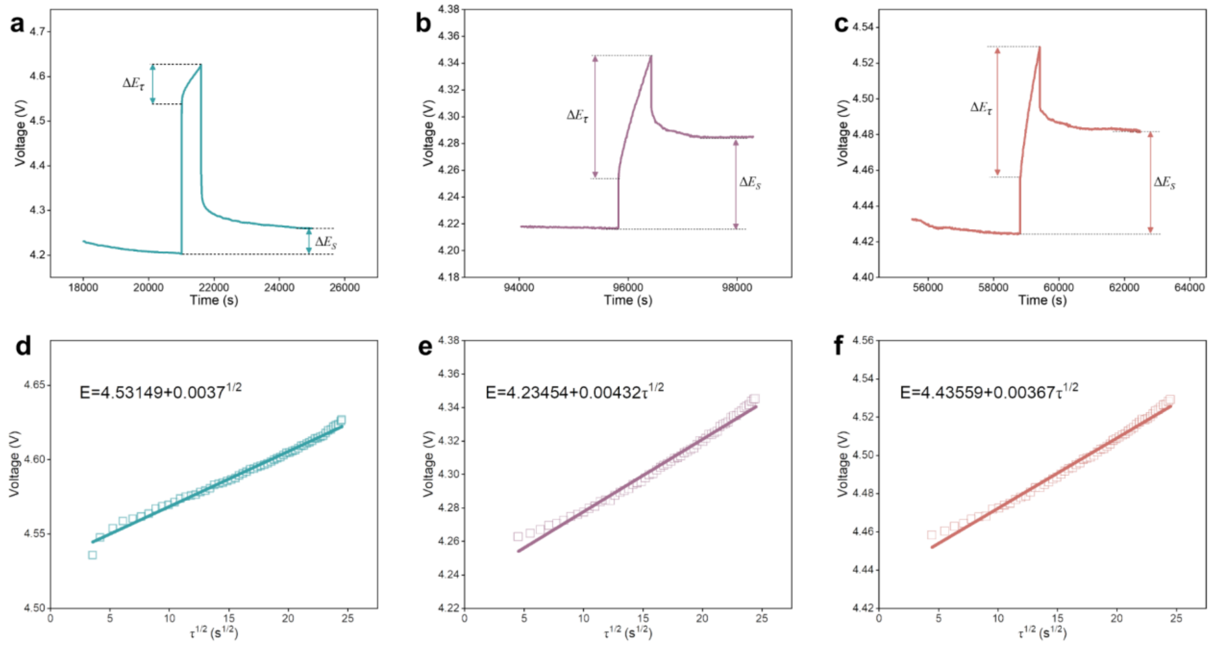

Fig. S14 A typical time versus potential profile of **a** LCO, **b** MAT-LCO and **c** CS-LCO. A linear relationship between potential and $\tau^{1/2}$ of **d** LCO, **e** MAT-LCO and **f** CS-LCO

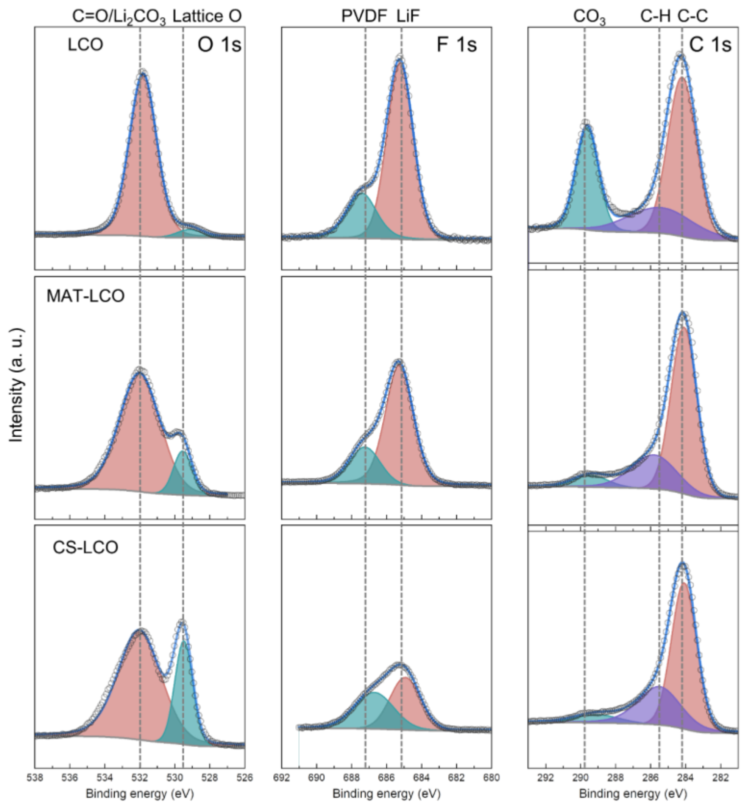

Fig. S15 The O 1s, F 1s and C1s XPS spectra of LCO MAT-LCO and CS-LCO cathode after 100 cycles

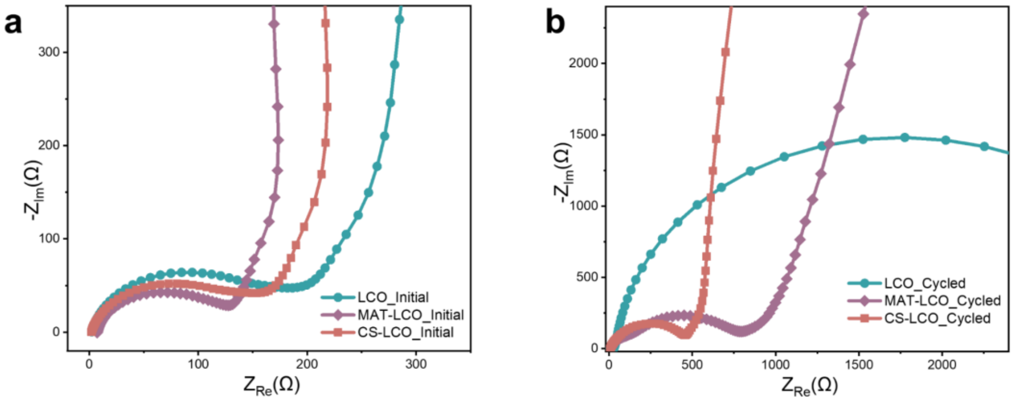

Fig. S16 The Nyquist plots of the LCO, MAT-LCO and CS-LCO electrodes: **a** initial cycle and **b** after 100 cycles

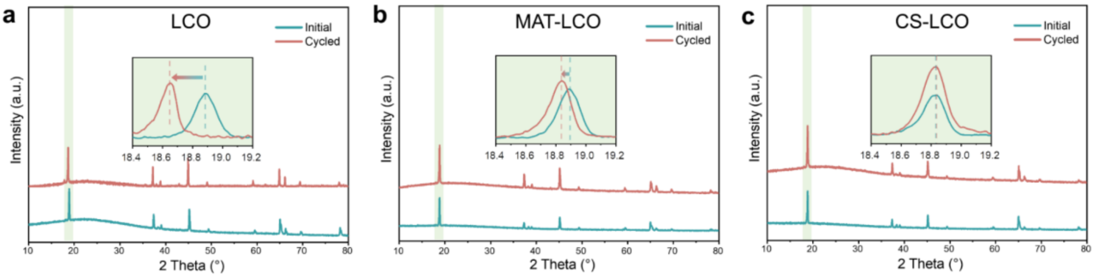

Fig. 17 Structural stability. The XRD patterns of **a** LCO and **b** MAT-LCO and **c** CS-LCO cathodes before cycling and after 100 cycles

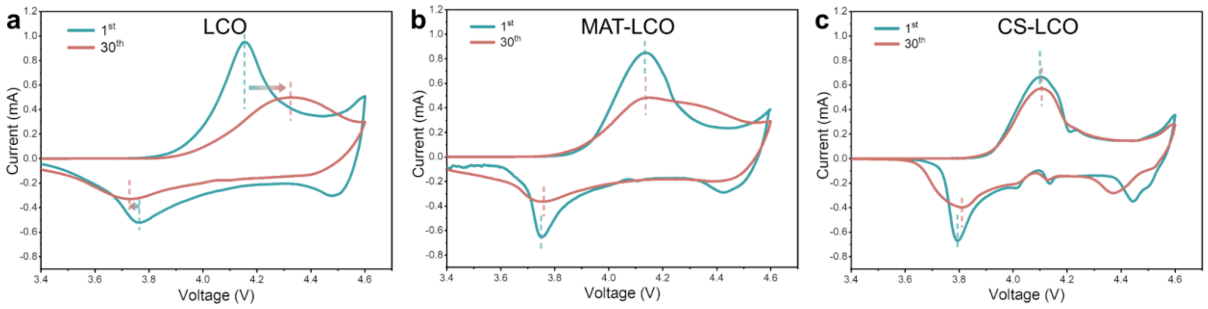

Fig. S18 The CV curves of **a** LCO, **b** MAT-LCO and **c** CS-LCO at initial cycle and after 30 cycles at 0.1 mV/s in half-cell configuration

Table S1 The simulated results from EIS spectra of LCO, MAT-LCO, and CS-LCO electrodes at various voltages during the initial charge/discharge cycle

Samples	Charge states	Simulated electrochemical parameters	
		Rsf (Ω)	*Rct* (Ω)
LCO	Charge to 3.2V	2488	1658
	Charge to 3.6V	1996	1564
	Charge to 4.0V	1761	1511
	Charge to 4.4V	424	774
	Charge to 4.6V	329	554
	Discharge to 4.2V	331	557
	Discharge to 3.8V	389	788
	Discharge to 3.4V	694	1437
	Discharge to 3.0V	1359	1483
MAT-LCO	Charge to 3.2V		364
	Charge to 3.6V		345
	Charge to 4.0V		237
	Charge to 4.4V	36	190
	Charge to 4.6V	32	70
	Discharge to 4.2V	41	79
	Discharge to 3.8V	37	190
	Discharge to 3.4V	167	237
	Discharge to 3.0V	706	263
CS-LCO	Charge to 3.2V	-	565
	Charge to 3.6V	-	571
	Charge to 4.0V	230	412
	Charge to 4.4V	186	318
	Charge to 4.6V	144	314
	Discharge to 4.2V	147	327
	Discharge to 3.8V	149	397
	Discharge to 3.4V	207	462
	Discharge to 3.0V	-	545
